# Serotonin receptor 4 in the hippocampus modulates mood and anxiety

**DOI:** 10.1038/s41380-020-00994-y

**Published:** 2021-01-13

**Authors:** Remzi Karayol, Lucian Medrihan, Jennifer L. Warner-Schmidt, Ben W. Fait, Meghana N. Rao, Eva B. Holzner, Paul Greengard, Nathaniel Heintz, Eric F. Schmidt

**Affiliations:** 1 Laboratory of Molecular Biology, The Rockefeller University, New York, NY 10065 USA; 2 Laboratory of Molecular and Cellular Neuroscience, The Rockefeller University, New York, NY 10065 USA; 3 NeuroJenic Consulting LLC, Garden City, NY 11530 USA; 4 Howard Hughes Medical Institute, The Rockefeller University, New York, NY 10065 USA

**Keywords:** Neuroscience, Molecular biology, Depression

## Abstract

Serotonin receptor 4 (5-HT_4_R) plays an important role in regulating mood, anxiety, and cognition, and drugs that activate this receptor have fast-acting antidepressant (AD)-like effects in preclinical models. However, 5-HT_4_R is widely expressed throughout the central nervous system (CNS) and periphery, making it difficult to pinpoint the cell types and circuits underlying its effects. Therefore, we generated a Cre-dependent 5-HT_4_R knockout mouse line to dissect the function of 5-HT_4_R in specific brain regions and cell types. We show that the loss of functional 5-HT_4_R specifically from excitatory neurons of hippocampus led to robust AD-like behavioral responses and an elevation in baseline anxiety. 5-HT_4_R was necessary to maintain the proper excitability of dentate gyrus (DG) granule cells and cell type-specific molecular profiling revealed a dysregulation of genes necessary for normal neural function and plasticity in cells lacking 5-HT_4_R. These adaptations were accompanied by an increase in the number of immature neurons in ventral, but not dorsal, dentate gyrus, indicating a broad impact of 5-HT_4_R loss on the local cellular environment. This study is the first to use conditional genetic targeting to demonstrate a direct role for hippocampal 5-HT_4_R signaling in modulating mood and anxiety. Our findings also underscore the need for cell type-based approaches to elucidate the complex action of neuromodulatory systems on distinct neural circuits.

## Introduction

Emotion is governed by a complex neural circuit comprised of anatomically and functionally distinct components that are distributed throughout the CNS. Functional imaging and postmortem analyses of brains of patients suffering from depression and/or anxiety disorders suggest a dysfunction in regions of the brain that control emotion, including limbic structures such as the hippocampus, amygdala, prefrontal cortex, and hypothalamus [1, 2]. Each of these areas is strongly modulated by the neurotransmitter serotonin (5-HT) while drugs that target 5-HT signaling, such as selective serotonin reuptake inhibitors (SSRIs), are a first-line treatment for affective disorders [3]. The widespread influence of 5-HT on numerous cognitive and behavioral processes leads to many unwanted side effects due to off-target engagement of AD drugs [4], and functional and anatomical separation of the neural substrates underlying pathology from those mediating therapeutic responses [5, 6]. This notion is reinforced by regional differences in the expression and regulation of genes and gene networks across the emotion circuit in disease-related contexts [7, 8]. Improved treatments for these diseases are dependent on a more precise understanding of cell types and molecular pathways mediating 5-HT-dependent signaling.

The 5-HT_4_ receptor (5-HT_4_R) is one of over 14 known 5-HT receptors in mammals and is strongly linked to AD responses [9]. 5-HT_4_R is an excitatory G_s_-coupled receptor that activates the cyclic AMP (cAMP)-PKA pathway and promotes the excitability of neurons [10, 11]. Changes in 5-HT_4_R binding were observed in several brain regions in depressed patients [12, 13] while polymorphisms in *HTR4* (the gene that encodes 5-HT_4_R) were associated with a susceptibility to unipolar depression [14]. In preclinical studies, short-term treatment with 5-HT_4_R agonists, including RS67333, has anxiolytic and antidepressant properties and mimics the cellular and molecular AD responses achieved after chronic SSRI administration [15–19]. In addition, 5‑HT_4_R agonists potentiate the effects of SSRIs [20] while constitutive *Htr4* knockout mice have reduced behavioral responses to acute stress and novelty [21]. 5-HT_4_R has also been linked to the regulation of adult neurogenesis in the dentate gyrus (DG) [16, 17] which is a reliable readout of AD efficacy and may underlie some therapeutic AD effects [22].

5-HT_4_R is highly expressed in many brain regions linked to mood and anxiety, namely the hippocampus, amygdala, prefrontal cortex, and striatum [10, 23], as well as peripheral tissues such as the gut, heart, and adrenal glands [24]. Such widespread expression in the CNS and periphery complicates the use of 5-HT_4_R as a viable therapeutic target for mood, as its activation can lead to significant gastrointestinal and cardiac complications [25, 26]. Most in vivo studies investigating 5-HT_4_R function have relied on systemic pharmacological approaches or constitutive deletion. Therefore, employing a cell type centered approach to elucidate distinct roles for 5-HT_4_R signaling in discrete circuits and/or cell types will facilitate the development of more precise and efficacious therapies.

To achieve this, we generated mice harboring a conditional mutant allele to delete functional 5-HT_4_R from genetically targeted cell populations expressing Cre recombinase. We used this line to investigate the role of 5-HT_4_R signaling in hippocampus, which plays a central role in affective behaviors [27, 28], and where 5‑HT_4_R is expressed at high levels [29]. We report that the loss of 5-HT_4_R specifically from excitatory neurons of the hippocampus led to AD-like effects at the behavioral, cellular, and molecular levels which were accompanied by an anxiogenic phenotype. Cell type-specific molecular profiling revealed that the ventral dentate gyrus (vDG) granule cells (GCs) underwent robust molecular adaptations in the absence of 5-HT_4_R. In addition, we observed enhanced neurogenesis in the vDG and reduced excitability of mature vDG GCs in the absence of 5-HT_4_R. Our data reinforce a direct role for hippocampal 5-HT_4_R in mediating affective behaviors, likely through the regulation of relevant intracellular pathways and neural excitability, and uncover an unexpected functional complexity within the hippocampal circuit due to the differential effect on depression- and anxiety-related behaviors.

## Results

### Generation and validation of an Htr4^Floxed^ mouse line

**Fig. 1:**
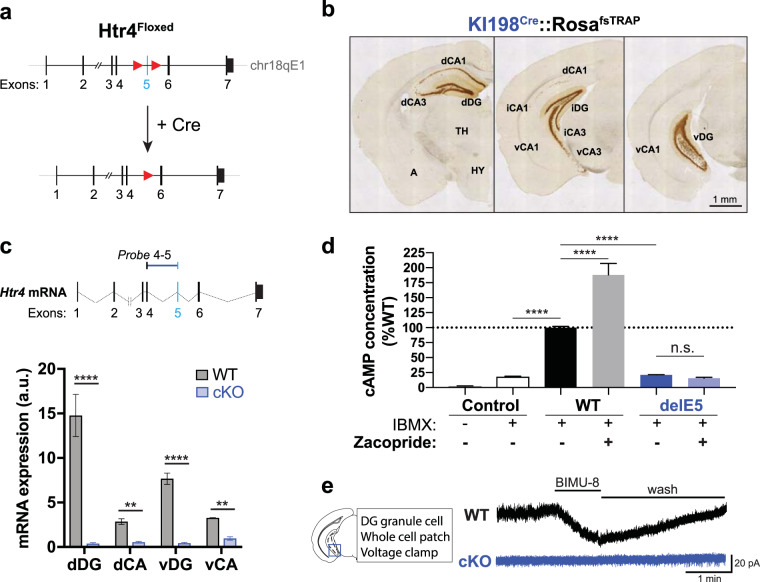
Generation and validation of an Htr4^Floxed^ mouse line. **a** Schematic of Htr4^Floxed^ allele. LoxP sites (red triangles) were inserted to flank *Htr4* exon 5 (light blue bar) for Cre-mediated excision. **b** Low magnification anti-EGFP immunohistology of coronal brain sections from a KI198^Cre^::Rosa26^fsTRAP^ mouse showing Cre expression in the hippocampus. dCA, iCA, vCA1/3: dorsal, intermediate, ventral CA1/3 fields, respectively; A: amygdala; TH: thalamus; HY: hypothalamus. **c** Schematic of the TaqMan probe spanning *Htr4* exons 4 and 5 (Probe 4–5) is shown at top. Below, qRT-PCR (mean ± SEM) quantification showing diminished expression of *Htr4* transcripts containing exon 5 along the dorsoventral axis of the hippocampus in Cre-negative (WT, gray) and cKO (blue) mice. Two-way ANOVA: genotype factor: F(1,24) = 439.0, *p* < 0.0001 followed by post hoc Fisher’s LSD test. *n* = 2–6 per group. **d** Quantification (mean ± SEM) of cAMP induction in HEK293T cells expressing EGFP (Control), intact 5-HT_4_R (WT), or *Htr4*^*delE5*^ (del5) in the presence or absence of the 5‑HT_4_R agonist, zacopride. One-way ANOVA followed by post hoc Fisher’s LSD test, *n* = 4 per group. **e** Representative traces of whole-cell voltage clamp recordings from DG GC in WT and cKO mice with bath administration of 10 mM BIMU-8. Scale bar: 1 min, 20 pA. Fisher’s LSD test, ****p < 0.0001, ***p < 0.001, **p < 0.01, n.s. *p* > 0.05.

To study the function of the 5-HT_4_R in specific cell types, we generated a novel mouse line (Htr4^Floxed^) to conditionally drive the deletion of functional 5-HT_4_R from genetically defined cell populations using the Cre/loxP system (Fig. 1a). Homologous recombination was used to insert loxP sites flanking exon 5 of the mouse *Htr4* gene, which encodes the fourth transmembrane domain of 5-HT_4_R (Fig. S1a and Document S1). In silico translation analysis predicted that the deletion of exon 5 would cause a frame shift mutation and the introduction of stop codons, including one right after the deletion site (Fig. S1b), leading to a truncated, unstable protein, that is unable to be inserted into the membrane, transduce a signal, and resulting in animals harboring a null *Htr4* allele.

Because *Htr4* is highly expressed in the hippocampal formation [29], we validated the deletion of exon 5 and the loss of functional 5-HT_4_R by generating a hippocampus-specific *Htr4* conditional knockout. The GENSAT BAC transgenic Drd3-Cre driver line KI198 (KI198^Cre^) was utilized since Cre recombinase expression was primarily limited to hippocampus [30]. The KI198^Cre^ did not fully recapitulate *Drd3* gene expression since the only visible expression outside of the hippocampus were a few scattered cells in ventromedial forebrain and islands of Calleja (www.gensat.org; Figs. 1b and S2b). Homozygous Htr4^Floxed^ mice were crossed to KI198^Cre^ mice to generate KI198^Cre^::Htr4^Floxed^ (cKO) mice (Fig. S1c). Quantitative RT-PCR (qRT-PCR) using probes spanning *Htr4* exons 4 and 5 (4–5) on RNA from dorsal and ventral subdivisions of the CA fields and DG revealed negligible expression of exon 5 in cKO samples compared to Cre-negative littermates (WT; Fig. 1c). In contrast, probes spanning exons 3–4 or 6–7 showed comparable expression between genotypes (Fig. S1e). These data confirm the generation of a mutant *Htr4* transcript lacking exon 5 (*Htr4*^*delE5*^) following Cre-mediated recombination in cKO mice.

Since 5-HT_4_R is a G_s_-coupled receptor that activates intracellular cAMP upon ligand binding [10], we employed a cell culture-based cAMP induction assay to test the signaling ability of the mutant receptor. Expression vectors for mutant *Htr4*^*delE5*^ (delE5) and intact *Htr4* (WT) were made by cloning each transcript from total RNA samples isolated from the hippocampus of a cKO mouse or a WT littermate, respectively. The constructs were transfected into HEK293T cells which were stimulated in the presence of IBMX, a phosphodiesterase inhibitor to allow accumulation of newly converted cAMP. Intact WT expression led to five-fold higher baseline levels of cAMP than of mutant delE5 or EGFP (control) (Fig. 1d). Stimulation with zacopride, a potent 5-HT_4_R agonist induced an 88% increase in cAMP production in cells expressing WT but did not significantly alter cAMP levels in those expressing delE5. These results suggest that exon 5 deletion leads to a non-functional receptor in vitro.

To test the functionality of mutant 5-HT_4_R in intact hippocampal neurons, we performed whole-cell voltage clamp recordings from DG GCs in acute hippocampal slices from cKO mice and WT littermates. The addition of the 5-HT_4_R agonist, BIMU-8, induced an inward current in WT GCs indicating a depolarizing effect due to the activation of 5‑HT_4_R (Fig. 1e), consistent with previous reports [11]. In contrast, cKO GCs did not respond to BIMU-8, validating the absence of functional 5-HT_4_R in DG GCs in cKO mice at the electrophysiological level. Together with the cAMP activation assay, these results indicate that the deletion of exon 5 in cKO mice resulted in a functionally null receptor. We did not notice an obvious spontaneous seizure phenotype in the cKOs.

### KI198^Cre^ expression is restricted to excitatory neurons in the hippocampus

**Fig. 2:**
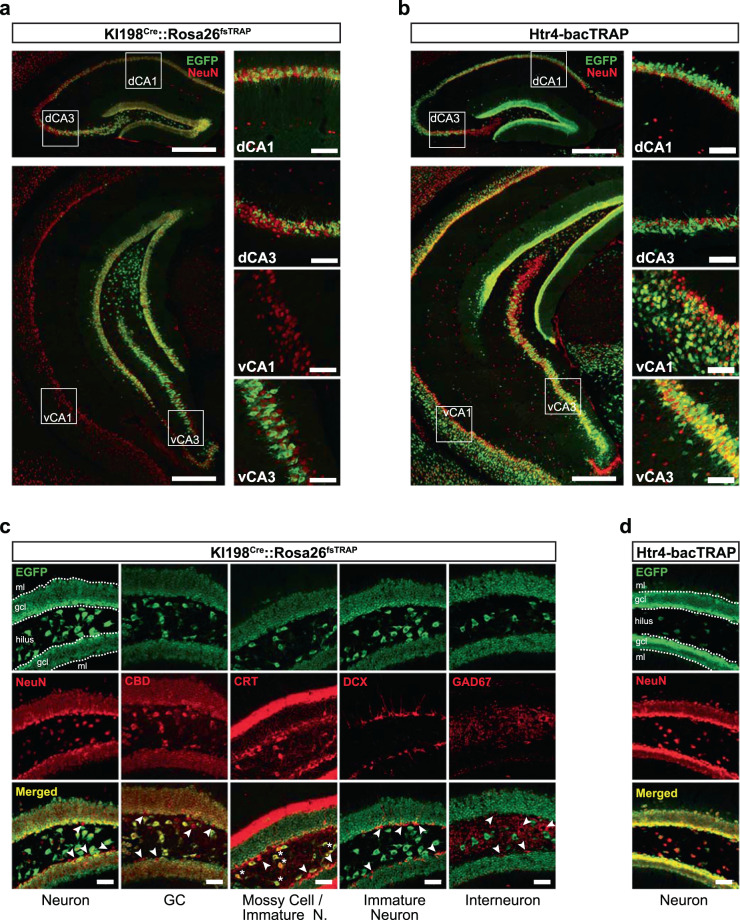
KI198^Cre^ expression is restricted to mature excitatory neurons in the hippocampus. **a** Anti-EGFP (green) and anti-NeuN (red) immunofluorescent confocal images of coronal hippocampal sections at the level of dorsal (top left) and ventral (bottom left) hippocampus from a KI198^Cre^::Rosa^fsTRAP^ mouse. Dorsal and ventral CA1 and CA3 fields (white boxes) are shown at higher magnification on the right. Scale bars: 500 µm (left panels) or 100 µm (right panels). **b** Anti-EGFP (green) and anti-NeuN (red) immunofluorescent confocal images of coronal sections at the level of dorsal (top left) and ventral (bottom left) hippocampus from an Htr4-bacTRAP mouse. Dorsal and ventral CA1 and CA3 fields (white boxes) are shown at higher magnification on the right. Scale bars: 500 µm (left panels) or 100 µm (right panels). **c** Immunofluorescent confocal images showing neuronal-type specific marker (red) and EGFP (green) expression in coronal sections through the DG of KI198^Cre^::Rosa^fsTRAP^ mice. Cells labeled with a neuron-type marker but not EGFP (arrowheads) and cells double-labeled with hilar mossy cell marker CRT and EGFP (asterisks) are indicated. ml: molecular layer, gcl: granule cell layer. Scale bars, 50 µm. **d** Immunofluorescent confocal images of coronal sections through the DG of Htr4-bacTRAP mice labeled with anti-NeuN (red) and anti-EGFP (green). ml: molecular layer, gcl: granule cell layer. Scale bars, 50 µm.

To determine the identity of the Cre-expressing cells in the hippocampus of KI198^Cre^ mice, we crossed them to the Rosa26^fsTRAP^ Cre-dependent reporter line [31], in which EGFP is fused to ribosomal protein L10a (EGFPL10a). Anti-GFP immunofluorescent staining of coronal brain sections from KI198^Cre^::Rosa26^fsTRAP^ mice revealed labeled cells along the dorsoventral axis of the hippocampus, including a subset of cells in dorsal CA1 (dCA1), dorsal CA3 (dCA3), ventral CA3 (vCA3), and most cells in the granule cell layer (GCL) and hilus along the dorsoventral axis of the DG (Fig. 2a, c). Few GFP-expressing cells were visible in ventral CA1 (vCA1). All GFP-expressing cells in CA fields were co-labeled with neuronal marker, NeuN, but not the GABAergic interneuron marker, glutamate decarboxylase (GAD67; Fig. S2c), suggesting KI198^Cre^ primarily labels excitatory pyramidal neurons in these regions. In DG, GFP was detected in all of the calbindin (CBD)-expressing mature GCs and calretinin (CRT)-expressing mossy cells (MCs) in the hilus (asterisks), whereas it was absent from doublecortin (DCX)- and CRT-expressing immature neurons in the GCL and GAD67-expressing GABAergic interneurons throughout the DG (Fig. 2d). Together, we conclude that KI198^Cre^ targets mature excitatory neurons in the DG, including GCs and hilar MCs, but not immature neurons, inhibitory interneurons, or non-neuronal cells.

To compare the distribution of KI198^Cre^ cells to 5-HT_4_R expressing cells in the hippocampus, we generated Htr4-bacTRAP mice where the EGFPL10a transgene was driven by a *Htr4* BAC promoter to recapitulate endogenous *Htr4* expression (Fig. S2a, b). GFP expression in the hippocampus of Htr4-bacTRAP mice was highly similar to KI198^Cre^ mice, primarily labeling pyramidal cells in the CA fields, and GCs and MCs in the DG (Figs. 2b, d and S2c, d). GFP was not observed in progenitor cells and immature GCs, suggesting a lack of 5-HT_4_R in these cells, as seen by others [32]. Htr4-bacTRAP labeled many more cells in the vCA1 and there was a notable lateral-to-medial gradient in the number of cells labeled in dCA3 and vCA3. This pattern was also seen by *Htr4* ISH (Fig. S2a and the Allen Brain Atlas, www.brain-map.org). Interestingly, KI198^Cre^ expression had an opposite CA3 gradient with denser labeling near the DG and fewer cells approaching CA2 (Fig. 2a, c). Since KI198^Cre^ reflects endogenous *Htr4* expression in the DG, crossing this line with Htr4^Floxed^ mice results in nonfunctional 5-HT_4_R throughout the DG. The incomplete overlap in the CA fields suggests only partial deletion of 5-HT_4_R function in this region. Notably GFP was visible in many other cells throughout the brain in the Htr4-bacTRAP mice, including cortex, hypothalamus, midbrain, and most cells in the ventromedial forebrain, contrasting with the restricted KI198^Cre^ expression (Fig. S2a, b).

### The effect of hippocampus specific loss of 5-HT_4_R on mood and anxiety related behaviors

**Fig. 3:**
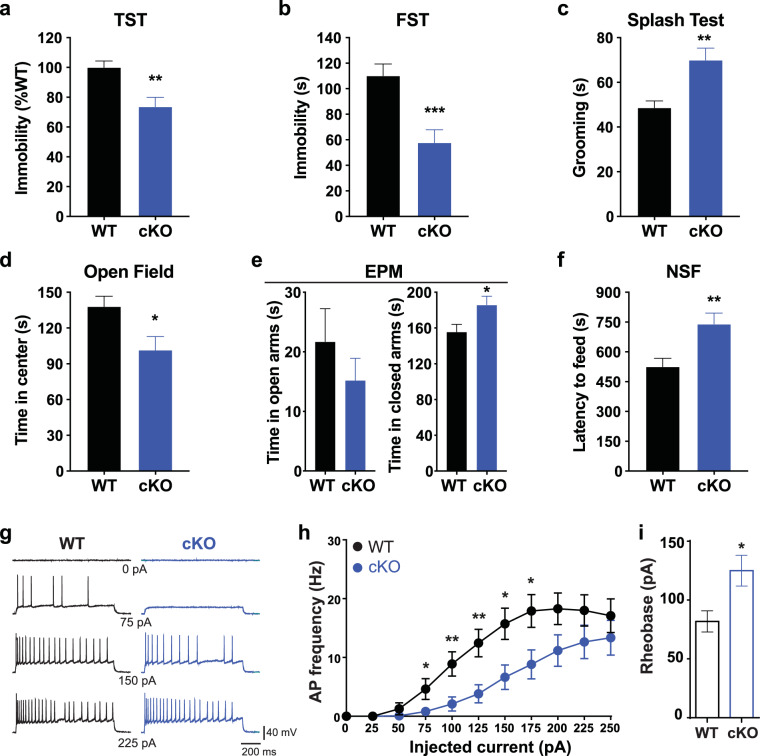
cKO mice exhibit altered affective behaviors and reduced firing in vDG granule cells. **a** Quantification of the time spent immobile in the tail suspension test (TST) in cKO mice (cKO, blue) and Cre-negative littermates (WT, black). **p = 0.0012, n_WT_ = 19, n_cKO_ = 16. **b** Quantification of the time spent immobile in the forced swim test (FST) for each genotype. ***p = 0.0005, n_WT_ = 19, n_cKO_ = 16. **c** Quantification of grooming time in the splash test for each genotype. **p = 0.001, n_WT_ = 19, n_cKO_ = 16. **d** Mean time spent in the center in the open field (OF) for each genotype. *p = 0.0139, n_WT_ = 19, n_cKO_ = 17. **e** Quantification of the time spent in the open (left) and closed (right) arms in the elevated plus maze (EPM). *p = 0.0239, n_WT_ = 18, n_cKO_ = 16. **f** Mean latency to feed in the novelty suppressed feeding (NSF) paradigm for each genotype. **p = 0.0033, n_WT_ = 20, n_cKO_ = 16. **g** Sample traces from whole-cell current-clamp recordings of GCs in hippocampal slices from WT and cKO mice showing spiking in response to different steps of injected current. **h** Quantification of the AP frequency of GCs in WT and cKO mice across current steps. **i** Histogram of rheobase measurements of GCs from WT and cKO mice. All data are represented as mean ± SEM and two-tailed unpaired *t*-tests were performed for all panels. *p < 0.05, **p < 0.01, ***p < 0.001.

Given the direct role of serotonin in the regulation of mood [33] and that 5-HT_4_R has been shown to mediate AD responses [16], we investigated the impact of the conditional ablation of 5-HT_4_R from hippocampus on mood-related behaviors. We first employed the tail suspension (TST) and forced swim tests (FST) which assess responses to an acute, inescapable stressor as a measure of behavioral despair [34–36]. Immobility was decreased in cKO mice compared to WT littermates in both TST and FST (Fig. 3a, b), indicating reduced despair-like behavior and mimicking an AD-like response. These results were not due to a general hyperactivity of the cKOs since they did not differ from WTs in locomotor behavior in an open field arena (Fig. S3e).

The splash test and sucrose consumption are commonly used as indicators of hedonic state in depression models and rely on the animal’s motivation for self-grooming and the rewarding properties of a sweet, palatable sucrose solution, respectively [37–39]. In the splash test, cKO mice spent more time grooming following the application of a sucrose solution compared to WT littermates, mimicking the effect of antidepressant administration (Fig. 3c). During the sucrose consumption, both cKO and WT mice drank approximately 5–10-fold more sucrose than water each day over the three-day test (Fig. S3a, b), implying no difference in hedonic capacity between genotypes. Interestingly, cKO mice consumed cumulatively more sucrose after 72 hrs compared to WT. This result was unlikely due to a change in caloric need since body weight was unaltered in the cKOs (Fig. S3c). The significance of the increased sucrose consumption is difficult to interpret given the strong sucrose preference exhibited by both groups but may reflect a subtle rise in pleasure-seeking drive. Together with the FST and TST results, these data indicate that cKO mice displayed reduced behavioral despair and increased hedonic drive, strongly mimicking an AD-like response.

As anxiety is closely associated with mood disorders and AD treatment, and has been linked to hippocampal and 5‑HT_4_R function [17, 40], we next assessed how hippocampus-specific loss of 5‑HT_4_R affected a variety of anxiety-related behaviors. The open-field test (OF), elevated plus maze (EPM) and novelty suppressed feeding (NSF) paradigms all rely on mice having an innate aversion to well-lit, open spaces while NSF adds the conflict of engaging in feeding behavior in a novel environment [41–44]. In the OF, cKO mice spent less time in the center of the arena during the initial 10 min compared to WT littermates while showing similar overall locomotor activity (Figs. 3d and S3d, e). The latency to enter the center also differed between groups (WT: 5.7 ± 2.2 s vs. KO: 20.4 ± 5.3 s; Mann–Whitney test: *U* = 78, *p* = 0.006) while total distance travelled in the center during the first 10 min showed a similar trend, although it did not reach statistical significance (WT: 661.6 cm ± 47.9 vs. KO: 538.8 cm ± 54.0; unpaired *t*-test: t(35)=1.706, *p* = 0.097). In the EPM, the cKOs spent more time in the closed arms compared to WT (Fig. 3e). Although the time spent in the open arms did not differ between genotypes, the cKOs displayed a trend towards higher velocity in EPM compared to WT (Fig. S3f) suggesting that higher exploratory activity of cKO mice was not accompanied by more exploration of the open arms. The cKO mice also displayed a greater latency to feed in NSF (Fig. 3f) yet consumed more food on average when placed back into their familiar home cages following NSF (Fig. S3g), conveying that the lack of motivation to feed was not the source of their prolonged latency. Together, these conflict-based behavioral analyses indicate that cKO mice have an anxiety-like phenotype.

We also tested behaviors that may be affected by anxiety levels such as social interaction and acoustic startle response (ASR). Mice were tested in the three-chamber social interaction test to measure sociability since a reduced amount of time spent interacting with a novel conspecific has been associated with abnormal anxiety-like behaviors [45]. We observed that cKO mice interacted less with a novel mouse compared to WT (Fig. S3h). Enhanced startle response has also been linked to increased anxiety and its physiological features [46, 47] while blunted startle was observed in patients with anhedonia [48–50]. We found that ASR was significantly enhanced in cKO mice compared to WT (Fig. S3i, j). On the other hand, the pre-pulse inhibition (PPI), the suppression of ASR when a startling stimulus is preceded by an immediate, weaker pre-stimulus, was not altered (Fig. S3k). Together, these anxiety-regulated responses reflect the anxiogenic phenotype observed in the cKOs.

### Loss of 5-HT_4_R reduces the excitability of dentate gyrus granule cells

Activation of 5-HT_4_R leads to depolarizing currents via second messenger mechanisms in excitatory cells of the hippocampus [11], including DG GCs (Fig.1d). To test the electrophysiological consequences of functional loss of 5-HT_4_R, we performed whole-cell current clamp recordings from vDG GCs in acute hippocampal slices from WT and cKO mice. We found that the firing frequency of GCs was significantly reduced in cKO slices compared to WT (Fig. 3g, h). In addition, rheobase, the amount of current necessary to be injected to elicit an action potential, was larger in GCs from cKO mice (Fig. 3i). On the contrary, there was no difference in the properties of single-action potentials between genotypes, including threshold, amplitude, half-amplitude width, and fast after-hyperpolarization as well as the input resistance and slow after-hyperpolarization (Fig. S4a-g). These data show that the loss of 5-HT_4_R leads to specific physiological changes resulting in a reduced excitability in DG GCs.

### Molecular adaptations in the vDG of cKO mice highlight cellular and functional roles for 5-HT_4_R

**Fig. 4:**
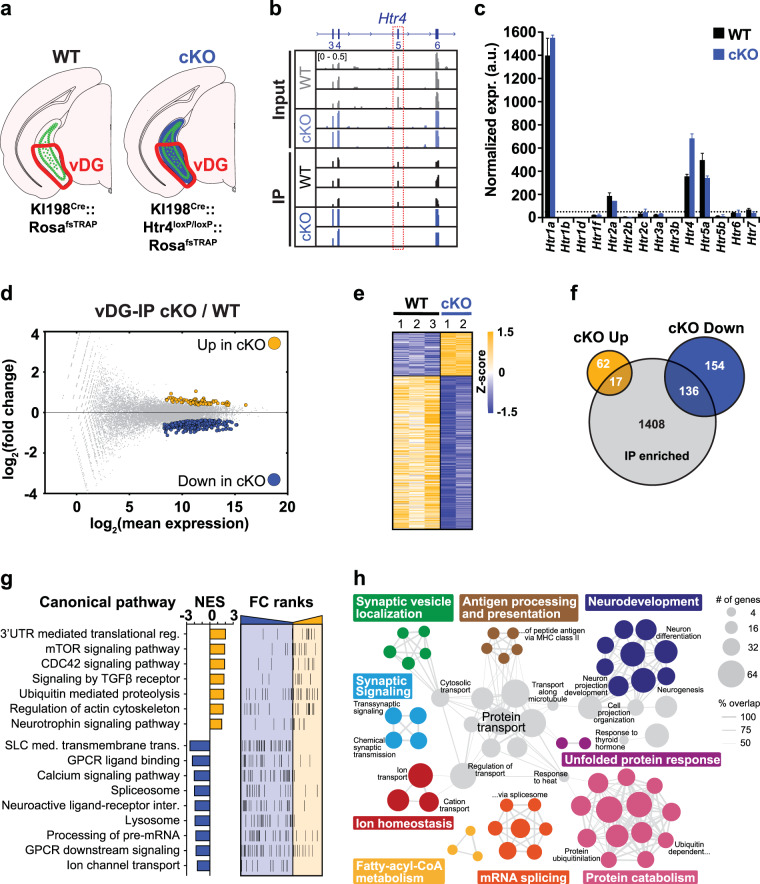
TRAP profiling of vDG neurons in the absence of 5-HT_4_R. **a** Schematic depicting the cell types targeted for TRAP. Green represents EGFPL10a expressing cells and blue indicates conditional 5-HT_4_R deletion. The area dissected is outlined in red. **b** Browser view of mapped reads showing the lack of expression of *Htr4* exon 5 in IP and input samples in the cKO. **c** Bar graph of normalized expression values (mean ± SEM) of all 5-HT receptors (Htrs) in IP samples. Dashed line is drawn at normalized expr. = 50. Only *Htr4* has FDR < 0.1. **d** MA-plot highlighting differentially expressed (DE) genes (FDR < 0.1) between the cKO and WT TRAP mRNA. Complete list of IP DE genes can be found in Table S1. **e** Heatmap visualization of DE genes between genotypes. **f** Venn diagram showing the overlap of up- and down-regulated DE genes from **e** with genes enriched in the KI198^Cre^ -expressing cells (IP enriched, Table S2). **g** Summary of gene set enrichment analysis (GSEA) performed on the fold change (FC)-ranked gene list (*p* < 0.05) between cKO and WT IP data sets. The normalized enrichment score (NES) shows the direction of enrichment in the cKO and FC-ranks are arranged from down-regulated (blue) at left to up-regulated (yellow) at right. Complete list of pathways can be found in Table S3. **h** Summary of gene ontology (GO) analysis of DE from **e**. All categories shown have FDR < 0.01, full analysis is provided in Table S4.

We next investigated global differences in gene expression that occurred following the deletion of hippocampal 5-HT_4_R to identify molecular mechanisms that may underlie the behavioral and electrophysiological phenotypes observed in cKO mice. We focused on the ventral DG since nearly all 5-HT_4_R expressing cells in the DG, but not CA fields, are targeted by the KI198^Cre^ line (Fig. 2a–c), and there is a strong link between ventral hippocampus and the regulation of depression and anxiety [27]. The translating ribosome affinity purification (TRAP) method [51, 52] was used to isolate cell type-specific transcripts by expressing the EGFPL10a TRAP transgene in WT and cKO KI198^Cre^ cells by crossing KI198^Cre^ with Rosa26^fsTRAP^, and KI198^Cre^::Htr4^Floxed^ with Rosa26^fsTRAP^::Htr4^Floxed^ mice, respectively (Fig. 4a). Polysome-bound mRNAs were extracted following anti-EGFP immunoprecipitations (IP) on vDG homogenates and analyzed by RNA-seq alongside mRNAs extracted from whole tissue (input) (Fig. S5a). The quality of dissection was confirmed by qRT-PCR which showed that markers for DG (*Dsp)* and vDG (*Trhr*) were highly expressed in vDG inputs but markers for CA fields (*Tyro*) and dDG (*Lct*) were not (Fig. S5b-c). Visualization of RNA-seq reads mapped to the *Htr4* locus confirmed the deletion of exon 5 in cKO tissue (Fig. 4b), although overall expression of *Htr4* was increased in cKO TRAP mRNA compared to WT, likely reflecting an attempt by the cells to compensate for the non-functional protein (Fig. 4c). There did not appear to be a broader compensation in 5-HT signaling since there was no difference in the expression of other 5-HT receptor transcripts between genotypes (Fig. 4c). Consistent with previous reports of the distribution of 5-HT receptors in the DG [29], the receptors with highest expression in the vDG TRAP were *Htr1a*, *Htr2a*, *Htr4*, and *Htr5a*.

A direct comparison of gene expression between TRAP mRNA (IP) and whole tissue mRNA (input) revealed a substantial depletion of markers for interneurons, progenitors, and other non-neuronal cells in the TRAP data set (Fig. S5d–f), consistent with our immunohistology showing the KI198^Cre^ line primarily labels mature GCs and hilar MCs in the DG (Fig. 2c). While markers for GCs were highly expressed in TRAP mRNA, their underwhelming levels of enrichment in IP over input, compared to the depletions of markers of cells not labeled by KI198^Cre^ (Fig. S5e, f), were likely because most of the transcripts in the input were from mature GCs since they constitute the vast majority of all cells in the DG. TRAP IPs from WT and cKO mice showed a similar distribution of marker genes and IP-enriched transcripts (Figs. S5e, f and S6a) indicating that the mutant allele did not noticeably alter cellular identity.

Differential expression analysis of TRAP mRNA identified 80 upregulated and 290 downregulated genes in cKOs compared to WT (Fig. 4d). Heatmap visualization based on normalized expression values showed that the significant gene expression differences between genotypes were consistent across all replicates (Fig. 4e). Over 40% of the differentially expressed (DE) genes (17 up- and 136 downregulated) were enriched in the IP of KI198^Cre^ cells (Fig. 4f), illustrating a strong cell type-specific adaptation. Quantitative RT-PCR (qRT-PCR) of selected genes confirmed these results showing that *Cox8a*, *Trib2*, *Trmt112*, and *Wbscr22* were enriched while *Cdh10*, *Pik3r3*, *Rdx*, and *Ryr2* were depleted in cKOs at similar levels to RNA-seq data (Fig. S5g). To gain a general understanding of the gene pathways dysregulated in the cKOs, we performed gene set enrichment analysis (GSEA) on a ranked list of genes differing between WT and cKO samples (Fig. 4g). We found positive enrichment in signaling pathways linked to translation, neuroplasticity, and cytoskeletal regulation, including mTOR, CDC42, TGFβ, neurotrophin signaling, and regulation of actin cytoskeleton, all of which have been shown to be regulated by AD treatment. Interestingly, there was an upregulation in ubiquitin proteolysis and 3’UTR mediated translation, and a downregulation in pre-mRNA processing, implying a possible response to the handling of the mutant *Htr4* transcript and 5-HT_4_R protein. We found negative enrichment in pathways related to 5-HT_4_R function including GPCR ligand binding, calcium signaling, neuroactive ligand-receptor interaction, and GPCR downstream signaling, paralleled by decreased expression of genes involved in cAMP signaling (Figs. 4g and S6b). In addition, there was a downregulation in pre-mRNA processing and transmembrane transport of various classes of ions (Figs. 4g and S6c). Functional annotation analysis focused on significantly DE genes between genotypes showed alterations in processes underlying neuronal function including synaptic vesicle localization, neuron development, synaptic signaling, and ion homeostasis, while unfolded protein response and protein catabolism imply a change in proteostasis (Fig. 4h). Together, our molecular profiling of the vDG from cKO and WT mice identified substantial transcriptional changes in the absence of 5-HT_4_R that are related to neuronal function and intracellular 5‑HT_4_R receptor signaling.

### The ablation of 5-HT_4_R resulted in enhanced adult neurogenesis in the vDG

**Fig. 5:**
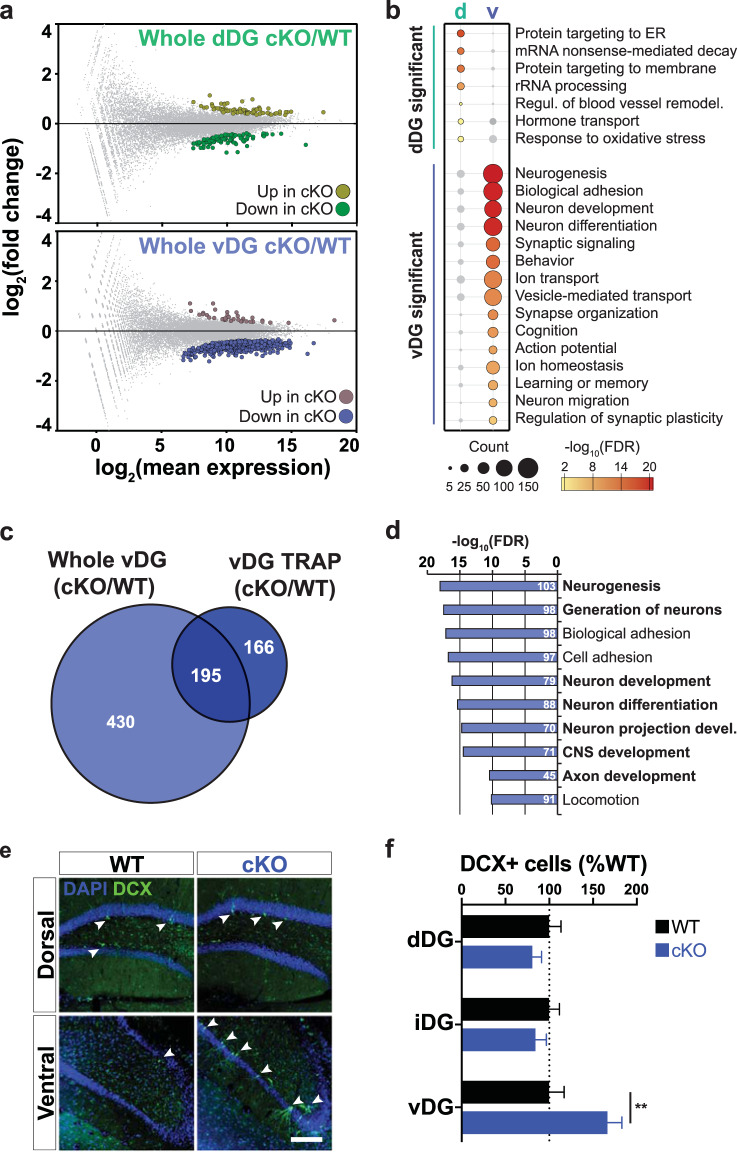
Loss of 5-HT_4_R led to changes in neurogenesis-related genes and increased number of immature neurons in the vDG. **a** MA-plots indicating differentially expressed (DE) genes (FDR < 0.05) between cKO and WT mRNA from whole dDG (top) and vDG (bottom). Complete lists can be found in Tables S5 and S6. **b** Comparison of significantly enriched gene ontology (GO) terms among whole dDG and vDG DE genes from **a**. **c** Venn diagram comparing DE transcripts shared and unique to vDG TRAP (dark blue) and whole vDG tissue (light blue) samples. **d** Top ten GO terms enriched in the whole vDG-specific gene list from **c**. Terms related to neurogenic tone are in bold. Complete lists can be found in Tables S7 and S8. **e** Representative images of anti-DCX immunofluorescence in coronal sections through dDG and vDG from cKO and WT mice. Arrowheads indicate DCX+ cells. DAPI is shown in blue. Scale bar, 150 µm. **f** Quantification (mean ± SEM) of DCX+ cells per granule cell layer area in the dorsal (dDG), intermediate (iDG), and ventral (vDG) DG. Two-way ANOVA: genotype × region interaction, F(2,24) = 6.328, *p* = 0.0062 followed by post hoc Fisher’s LSD test, **p = 0.002. *n* = 5 per genotype.

Up to this point, we have investigated the effects of 5-HT_4_R loss on the intrinsic molecular and electrophysiological properties *Htr4*-expressing cells. To ask how these adaptations impacted the overall cellular environment within the DG, we utilized the whole tissue input mRNA since it was enriched with transcripts expressed in non-neuronal cells, progenitors, and interneurons (Fig. S5e), allowing us to examine broad molecular responses in non-*Htr4*-expressing cells. DE analysis between WT and cKO input tissue revealed a robust molecular response (625 DE genes) in the whole vDG and a relatively small response in the whole dDG (179 genes) (Figs. 5a and S7a). Only 25 genes were shared between regions (Fig. S7b, c). Functional annotation of DE genes revealed remarkably distinct cellular adaptations between the two regions. Processes related to neural function and plasticity, including neurogenesis, neuron development and differentiation, synaptic signaling, ion transport, and homeostasis, and regulation of action potential were uniquely enriched in the whole vDG mRNA but not dDG (Figs. 5b and S7d). The genes altered in the whole dDG mRNA were primarily related to protein and RNA processing. Furthermore, disease-gene association analysis of DE genes in the whole vDG also showed significant enrichment for a variety of diseases, including affective disorders such as depressive, bipolar, and anxiety disorders, along with other psychiatric disorders (Fig. S7e). No significant disease-gene associations were found in the dDG. In addition, 430 genes were uniquely altered in the vDG input but not TRAP IPs (Fig. 5c) reflecting changes that occurred primarily in non-*Htr4*-expressing cells. These genes were predominantly related to neurogenesis and neurodevelopment (Fig. 5d) whereas many genes unique to the IPs reflected intrinsic cellular processes such as ion homeostasis and transport, synaptic signaling, and protein processing (Figs. 5e, f and S6b).

Our genome-wide transcriptional analysis revealed significant alterations in gene expression related to neurogenesis and development in the whole vDG and an upregulation of factors known to promote neurogenesis and plasticity specifically in the KI198^Cre^ cells (Figs. 4e, f and 5b, d). This is particularly interesting given that the cKOs exhibit AD-like behavioral responses and the link between adult neurogenesis of DG GCs and SSRI antidepressants [27]. We therefore examined whether we could detect any change in hippocampal neurogenesis in the cKOs by quantifying the number of doublecortin-positive (DCX+) immature neurons along the dorsoventral axis of the subgranular zone (SGZ) and GC layer of the DG in WT and cKOs (Figs. 5e and S7f). There was a 67% increase in the number of DCX+ neurons in the vDG of cKO mice compared to WT littermates, while no difference in the number of DCX+ neurons was detected in the dDG or intermediate DG (Fig. 5f). These results suggest that loss of functional 5‑HT_4_R led to an increase in immature GCs in the vDG, which likely occurred through a non-cell-autonomous manner since KI198^Cre^ does not target progenitor cells or immature neurons. Together, these data demonstrate a remarkable amount of region-specific cellular and molecular adaptations along the dorsoventral axis of DG in response to loss of 5-HT_4_R function.

## Discussion

In the current study, we generated conditional 5-HT_4_R knockout mice to examine the role of this receptor in discrete cell populations in the hippocampal formation at behavioral, cellular, and molecular levels. Cre-mediated recombination resulted in a truncated and unstable protein as demonstrated by the absence of cAMP activation or an electrophysiological response to 5-HT_4_R agonists and a downregulation of 5-HT_4_R-related signaling pathways in cells harboring the mutant allele. The loss of 5-HT_4_R primarily from excitatory neurons in the DG and hilus led to AD-like performance in behavioral tests of despair and anhedonia, and elevated anxiety. DG GCs were less excitable and showed altered expression of genes related to GPCR function, ion transport, and neuroplasticity in the absence of 5-HT_4_R. In addition, cKO mice showed increased number of immature GCs in the vDG but not dDG, indicating regional differences in adaptions to 5-HT_4_R loss in the local environment along the dorsoventral axis. Taken together, these results strongly suggest that the congenital removal of functional 5-HT_4_R from excitatory cells in the DG leads to cellular and molecular adaptations that mimic chronic AD treatment and offers insight into the modulation of mood by hippocampal circuits.

Decreased functionality of hippocampal 5-HT_4_ receptors is consistent with reports describing cellular responses to antidepressants. Chronic SSRI treatment has been shown to reduce the density of 5-HT_4_R binding and decrease 5-HT_4_R-dependent cAMP signaling and postsynaptic excitability in hippocampal pyramidal cells in rats [53]. Moreover, short-term treatment with the partial 5-HT_4_R agonist, RS67333, led to 5-HT_4_R desensitization in hippocampus [18]. These findings together with our results highlight a multifaceted role for 5-HT_4_R in the regulation of hippocampal circuits underlying mood. 5-HT_4_R is expressed in many limbic structures besides the hippocampus including prefrontal cortex, ventral striatum, and amygdala [19] and it is generally thought that 5-HT_4_R activation is therapeutic. Indeed, the mPFC has been implicated as a site mediating the fast-acting anxiolytic and/or antidepressant effects since RS67333 [15, 54] and the overexpression of 5-HT_4_R specifically in the mPFC led to AD-like behavior [55]. We previously found that chronic SSRI administration led to increased *Htr4* expression in corticostriatal neurons in sensorimotor cortex [5], although it does not appear to underlie SSRI-mediated changes in 5-HT responses in those cells [56]. The lack of impact of constitutive deletion of 5-HT_4_R on baseline mood [21] further attests to the complex interplay between different circuit components that express the receptor. We cannot rule out that 5-HT_4_R deletion from the subset of CA pyramidal cells that overlap in *Htr4* and KI198^Cre^ expression or the scattered KI198^Cre^-expressing cells in the ventromedial forebrain and islands of Calleja contributes to the observed behavioral phenotypes, given their role in mood [57, 58]. However, the nearly complete overlap of KI198^Cre^ and *Htr4* in DG and the discernable molecular and electrophysiological adaptations in the vDG GCs of cKO mice strongly support a fundamental role for hippocampal 5-HT_4_R.

The reduced electrophysiological excitability of DG GCs in the absence of 5-HT_4_R was either directly due to a deficiency of 5-HT_4_R signaling or a result of long-term cellular adaptations in the absence of the receptor. 5-HT_4_R has been shown to contribute to the excitability of pyramidal cells through PKA-mediated closure of Ca^2+^-activated K^+^ channels underlying sAHP current [11, 59–61], however, we did not see any change in slow or fast AHPs in the cKOs. A direct coupling to both voltage-sensitive calcium channels [62] and the extracellular signal-regulated kinase pathway [63], both of which are known to modulate neuronal function [64, 65], has also been proposed for 5-HT_4_R function. The TRAP data revealed a dysregulation of Ca^2+^ signaling, ion homeostasis, and/or ion channel expression in the cKOs, although more work is needed to understand the significance of these changes on GC excitability.

The decreased excitability of the vDG GCs may have directly contributed to the AD-like phenotype of the cKOs. Growing evidence supports a link between suppressed activity of DG GCs and AD-like behavior. Decreased excitability of vDG GCs through local GABAergic inhibition produces behavioral AD action [66], and indirect inhibition of mature GCs of the vDG by immature GCs, or by chemogenetic methods promotes resilience to stress [67]. In addition, SSRI-mediated inhibition of GCs through 5-HT_1A_R is necessary for AD responses in preclinical models [68]. Blockade of GC-CA3 glutamate release and NMDA receptor activity reduces behavioral despair [69], and the therapeutic effects of antidepressants and 5-HT_4_R agonists in rodents are dependent on the tonic inhibition of CA3 pyramidal cells via the activation of 5-HT_1A_ receptors [16]. Finally, chronic AD administration attenuated excitatory physiological effects of 5-HT_4_R throughout the hippocampal circuit [53, 70]. It is possible that the 5-HT_4_R loss led to a shift in the balance of excitatory and inhibitory 5-HT signaling in DG which contributed to the AD-like phenotype of the cKOs. Both 5-HT_4 _and 5-HT_1A_ receptors are highly expressed in DG GCs and cooperate to mediate cellular responses to 5-HT despite activating opposing second messenger pathways and electrophysiological properties [9, 68]. The broad downregulation of cAMP-related genes and GPCR signaling detected in the cKO TRAP data support this idea.

The anxiogenic phenotype of the 5-HT_4_R cKO mice reinforces a role for hippocampal 5-HT_4_R in directly moderating anxiety, possibly though modulating the excitability of vDG GCs. Acute optogenetic activation of vDG GCs was shown to suppress innate anxiety, although increased anxiety was not observed when vDG GCs were acutely inhibited [40]. Long-term inhibition of GCs due to chronic loss of 5-HT_4_R functionality in the cKOs may have amplified this effect in our study. Systemic administration of pharmacological agents have also demonstrated robust anxiolytic properties of 5-HT_4_R agonists while 5-HT_4_R antagonists blocked the anxiolytic effects of diazepam and SSRIs [17, 19, 71]. Discrete subsets of vCA1 projection neurons also control anxiety-related behaviors depending on their postsynaptic targets [72, 73], indicating a complexity of cell-specific mechanisms even within the hippocampal circuit. Although KI198^Cre^ was absent from vCA1, likely preserving 5-HT_4_R function in most vCA1 pyramidal cells in the cKOs, reduced activity of vDG GCs probably had a significant impact on the trisynaptic hippocampal circuit and vCA1 outputs.

5-HT_4_R deletion also had a palpable effect on molecular and cellular neuroplasticity in the vDG. Cell type-specific molecular profiling of vDG GCs with TRAP provided evidence for an alteration in the neuroadaptive state of the cells highlighted by a change in genes linked to processes such as neuronal development, differentiation, and synaptic signaling. These pathways are differentially regulated in the hippocampus with AD treatment, deep brain stimulation, and exposure to stress [7, 8, 74–77] and likely underlie the changes in synaptic plasticity, spine density, and dendritic architecture of hippocampal neurons observed after AD administration [3, 78, 79]. The molecular adaptations in GCs were accompanied by an increase in the number of immature GCs selectively in the vDG in the cKOs. Adult hippocampal neurogenesis mediates some of the behavioral effects of SSRI treatments and responses to stress [37, 67, 80] but does not produce AD-like behavior on its own [81]. The absence of 5-HT_4_R and Cre from neural progenitors and immature GCs suggests the enhanced neurogenesis was a response to changes in the local environment, although based on our results we cannot definitively conclude whether the increase in immature GCs was due to changes in proliferation, survival, or maturation. We speculate that the cKOs have a deficit in maturation given the dysregulation of genes related to neurodevelopment and differentiation together with previous reports showing no change in baseline proliferation in global KOs [32] and the promotion of survival after pharmacological stimulation of 5-HT_4_R in hippocampus [17]. The lack of change in neurogenesis in the dDG underscores the functional divergence of the DG along the dorsoventral axis [27, 82] and the regional specificity of 5-HT_4_R function. A similar anatomical discrepancy in neurogenesis was observed in patients and mice following chronic SSRI treatment [83, 84] and the ablation of adult-born GCs in the vDG, but not dDG attenuated some of the behavioral responses to AD treatment, stress, and chronic pain [85, 86].

The present study begins to refine a role for 5-HT_4_R in a key circuit that mediates mood and anxiety and reinforces the complexity of neuromodulatory signaling in arbitrating emotion. This notion is underscored by the fact that the AD-like phenotype following the cell type-specific conditional deletion of 5-HT_4_R was accompanied by an increase in anxiety. SSRIs and other AD drugs are commonly used to treat anxiety disorders so the anxiogenic behavior of the KI198^Cre^ 5-HT_4_R cKO mice indicates a dissociation between the mechanism underlying these behaviors. Indeed, antidepressants can promote suicidality, anxiety, and nervousness, posing clinical concerns, and acute and chronic SSRI treatment in rodents can induce anxiety-like behaviors depending on the baseline anxiety/stress/depression levels, strain, age, dose, and delivery method of AD treatment [87–90]. Our data justify further inquiry to determine if these phenomena are moderated by a direct result of chronic disengagement of hippocampal 5-HT_4_R or due to an indirect effect, such as compensatory cellular and circuit-level adaptations resulting from non-functional receptor throughout development. Such observations and the widespread impact of the therapeutic targeting of broad neuromodulatory systems underscore the need to tease apart regional, temporal, and cell type-specific mechanisms to develop better drugs with diminished side effects.

## Materials and methods

### Animals

All procedures involving animals were approved by The Rockefeller University Institutional Animal Care and Use Committee and were in accordance with National Institutes of Health guidelines. KI198^Cre^ (Drd3-Cre KI198) mice were generated by the GENSAT Project [30] and were purchased from the Mutant Mouse Regional Resource Center (MMRRC) Repository (Stock #031741-UCD). Rosa26^fsTRAP^ mice were purchased from the Jackson Laboratories (Stock #022367). Htr4^Floxed^ and Htr4-bacTRAP mice were generated as described in Supplementary Methods. All animals were bred on a C57BL/6 J background, group-housed, maintained on a 12-h light-dark cycle at The Rockefeller University, and given ad libitum access to food and water. Animals used in the study were male, except for RNA sequencing experiments for which samples from male and female mice were pooled.

### Behavior

Behavioral tests were performed on male mice from 8 weeks to 4 months of age. For each test, mice were age-matched, and controls consisted of Cre-negative littermates. For each test, mouse order was randomized, and the experimenter was blinded to genotype during the tests and data analyses. All tests were performed within the light period of the light-dark cycle unless otherwise noted. The number of animals per group (*n*) and the statistical analyses are reported in the figure legends. All behavior tests were performed using established, published protocols. See Supplementary Methods for details.

### Total RNA isolation and quantitative RT-PCR (qRT-PCR)

Total RNA was isolated from dissected tissue using RNeasy Micro Kit (Qiagen, Germantown, MD). cDNA samples were generated using qScript cDNA SuperMix (QuantaBio, Beverly, MA). All qRT-PCR experiments were performed on the LightCycler 480 System (Roche Diagnostics, Indianapolis, IN), using TaqMan assays (Applied Biosystems, Foster City, CA). Three technical triplicates were run, and the mean C_T_ was used for the quantification for each sample. Data were normalized to *Gapdh* by the comparative C_T_ (2^-ΔΔCT^) method. See Supplementary Methods for details.

### cAMP induction assay

Intact *Htr4* (WT) and mutant *Htr4*^*delE5*^ (delE5) cDNA coding sequences (CDSs) obtained from the DG of cKO and WT mice were cloned into a mammalian expression plasmid as described in Supplementary Methods. HEK293T cells were transfected with the delE5, WT, or EGFP plasmids. Forty-eight hrs after the transfection, cells were incubated in stimulation buffer consisting of DMEM + 0.5 mM IBMX (Sigma Aldrich, St. Louis, MO) for 30 min. For cAMP induction, cells were further incubated in either stimulation buffer or 100 µM Zacopride (Tocris Bioscience, Bio-Techne, Minneapolis, MN) in stimulation buffer for 45 min. cAMP levels in the supernatants were measured by monoclonal anti-cAMP antibody-based direct cAMP ELISA kit according to the manufacturer’s guidelines (NewEast Biosciences, King of Prussia, PA). Four biological replicates and two technical replicates were performed for each experimental group. The cAMP level of each biological replicate was normalized to its mean protein concentration measured using Qubit protein assay kit (Thermo Fisher Scientific, Waltham, MA). See Supplementary Methods for detailed description.

### Immunohistochemistry

Immunohistochemistry and immunofluorescent staining were carried out on free-floating brain sections and imaged on either a Zeiss LSM700 confocal microscope or Zeiss Axioskop 2 microscope. Brightness was optimized using ImageJ software post-acquisition. For the quantification of neurogenesis, an experimenter blinded to genotype counted number of DCX-positive (DCX+) cells in the DG granule cell layer (GCL) in every sixth 40 µm thick coronal section through the entire hippocampus of WT or cKO mice. Neurogenesis was calculated as the number of DCX+ cells per area of GCL within a subdivision and values for each animal (cKO and WT) was normalized to the WT mean for each subdivision. For details on experimental procedures and antibodies used see Supplementary Methods.

### Fluorescent in-situ hybridization (FISH)

The RNAscope^®^ Multiplex Fluorescent Reagent Kit V2 (Advanced Cell Dagnostics, Newark, CA) was used for *Htr4* FISH. See Supplementary Methods for details.

### Translating ribosome affinity purification (TRAP)

Affinity purification of EGFP-tagged polysomes and RNA extraction were performed as previously described [51] with minor modifications as detailed in Supplementary Methods. Biological replicates consisted of relevant brain tissue pooled from one male and one female mouse.

### RNA sequencing (RNA-seq)

RNA was amplified using the Ovation RNA-Seq System V2 Kit (NuGEN, Redwood City, CA) and RNA-seq libraries were prepared using the TruSeq RNA Sample Preparation Kit v2 (Illumina, San Diego, CA) following manufacturer’s protocols. Libraries were sequenced at The Rockefeller University Genomics Resource Center on the Illumina NextSeq 500 platform. RNA-seq reads were aligned to annotated exons using the mm10 mouse reference genome (UCSC) with STAR (2.4.2a) [91]. The quantification of aligned reads was done using HTSeq framework (0.6.0) [92]. Differential expression analysis was performed using DESeq2 [93]. Differentially expressed (DE) genes for each analysis were determined based on adjusted *p* values (p-adj) and reported in the Results section or Figure Legends. See Supplementary Methods for further details.

### Electrophysiological recordings

Mice of 8 weeks were used for slice electrophysiology. Slice preparation, data acquisition, and analysis were performed with experimenter blinded to mice genotype. See Supplementary Methods for detailed description.

### Statistics

All statistical analysis was performed using GraphPad Prism 7 and 8, Microsoft Excel or R. Sample size was based on prior studies [5, 19]. Statistical parameters including the exact value of *n*, precision measures (mean ± SEM) and statistical significance are reported within the Results or the Figure Legends. Data were determined to be statistically significant when *p* < 0.05 by two-way ANOVA (ordinary or repeated measures [RM]) followed by post hoc Fisher’s LSD test, one-way ANOVA followed by post hoc Fisher’s LSD test, or two-tailed unpaired *t*-test. All data met the assumptions of applied statistical tests. Statistical methods to analyze RNA-seq data are detailed in Supplementary Methods.

## Supplementary information

Supplemental Information

Document S1

Table S1

Table S2

Table S3

Table S4

Table S5

Table S6

Table S7

Table S8

Table S9

## Data Availability

RNA-seq data sets are available from the NCBI Gene Expression Omnibus series accession number GSE157607.
