# Supplementary material for: Serotonin receptor 4 in the hippocampus modulates mood and anxiety
Source: Mol Psychiatry. 2021 Jan 13;26(6):2334–49. doi: 10.1038/s41380-020-00994-y (PMC8275670; doi:10.1038/s41380-020-00994-y)
Supplement: Supplementary file 1 — Supplemental Information [file 41380_2020_994_MOESM1_ESM.docx]

**Serotonin receptor 4 in the hippocampus modulates mood and anxiety**

***Supplemental Information***

**Supplemental Figures:**

(next page)

**
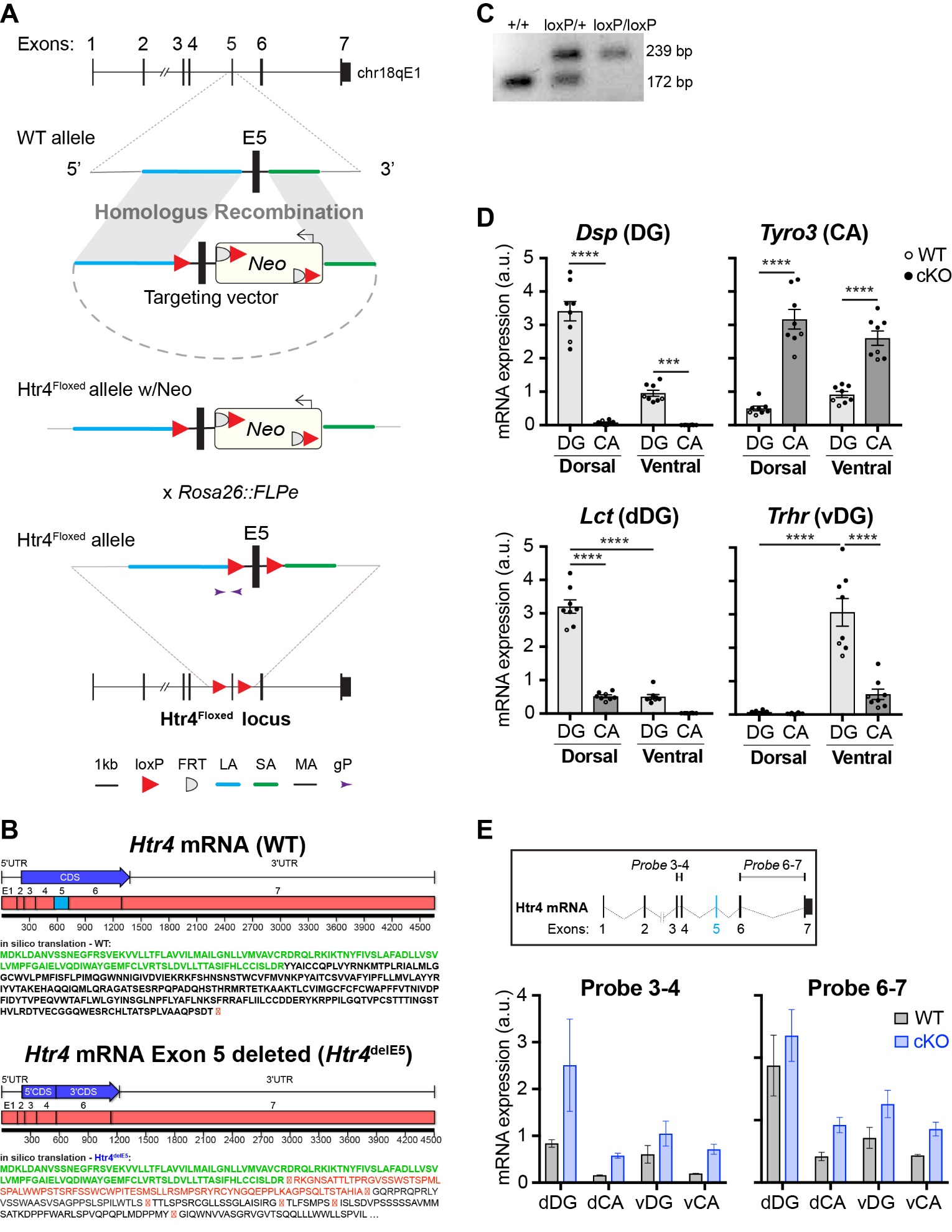
Fig. S1 | Gene targeting strategy to generate Htr4^Floxed^ mouse line.** Related to Fig. 1. **a**, Schematic of *Htr4* gene targeting steps. Targeting vector was designed to drive the homologous recombination via long (LA, blue) and short (SA, green) arms to insert the loxP-flanked exon 5 (vertical black bar) and Neomycin resistance cassette (Neo, yellow rectangle). Relative positions of FRT (flippase recognition target) sites (grey half circle) and loxP sites (red triangle) are shown. FLPe-mediated recombination was used to excise the Neo cassette and lead to *Htr4* exon 5 flanked with loxP sites (Htr4^floxed^ allele). gP, genotyping primers (purple arrowheads). Scale bar for magnified schematics, 1kb. **b**, In silico translation analysis predicting that the loss of exon 5 from *Htr4* mRNA would lead to immediate early stop codons. Green amino acid sequence represents the translation up to exon 5. Red squared X represents the translation of the stop codons. **c**, Sample genotyping results using gP from mice with wildtype (+/+), heterozygous (loxP/+) or homozygous (loxP/loxP) for the floxed insert showing the shift in amplicon size in the targeted allele. **d**, Quantitative RT-PCR (mean ± SEM) of the expression of marker genes for each hippocampal region in all samples used in **Fig. 1c**. Data points represent biological replicates of WT (empty circles, n = 2) and cKO (filled circles, n = 6) samples. One-way ANOVA followed by post hoc Fisher’s LSD test, ***p < 0.001, ****p < 0.0001. **e**, Top shows a schematic of TaqMan probes spanning *Htr4* exons 3-4 (Probe 3-4), and 6-7 (Probe 6-7). Bottom is quantitative RT-PCR results (mean ± SEM) showing the persistent expression of exon 3, 4, 6 and 7 in *Htr4* mRNA in cKO mice compared to WT along the dorsoventral axis of the hippocampus.

**
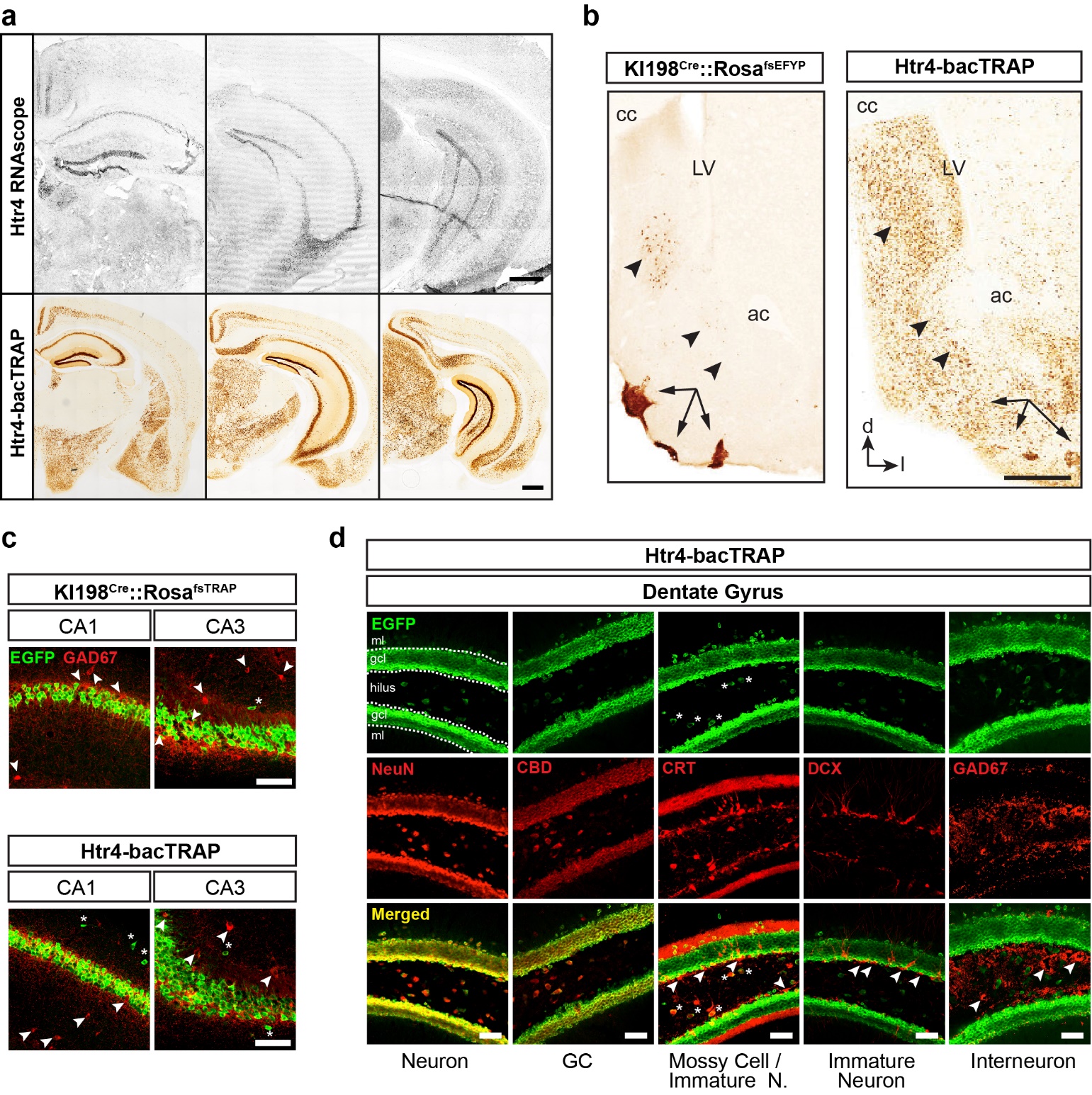
Fig. S2 | KI198^Cre^ and Htr4-bacTRAP expression is restricted to mature excitatory neurons in the hippocampus.** Related to Fig. 2. **a**, FISH images showing the spatial expression profile of *Htr4* in the mouse brain (top) and Anti-EGFP DAB immunohistochemistry of a Htr4-bacTRAP mouse brain (bottom) showing that the expression of the BAC transgene EGFPL10a under the regulatory elements of *Htr4* recapitulate the expression profile of *Htr4* in the brain. **b**, Anti-EGFP immunohistology showing Cre recombination in very few scattered cells in the septum (upper arrowhead) and nucleus accumbens (lower arrowheads) in the KI198^Cre^ line crossed an EGFP reporter (left panel) compared to widespread distribution of 5-HT_4_R expressing cells in those regions detected in the Htr4-bacTRAP line (right panel). Recombination was observed in the Islands of Calleja (arrows). ac, anterior commissure; cc, corpus collosum; LV, lateral ventricle. Compass show direction for dorsal (d) and lateral (l). Scale bar 500 µm. **c**, Anti-EGFP (green) and anti-GAD67 (red) immunofluorescent confocal images of coronal hippocampal sections from a KI198^Cre^::Rosa^fsTRAP^ (top) or Htr4-bacTRAP (bottom) mice. In both lines, EGFP expressing cells were GAD67-negative in both the CA1 (left), and CA3 (right) fields. Arrowheads, GAD67-postive; asterisks, GAD67-negative. Scale bar, 100 µm. **d**, Immunofluorescent confocal images showing neuronal-type specific markers (red) and EGFP (green) expression in coronal DG sections of Htr4-bacTRAP mice. EGFP was detected in all of the calbindin (CBD)-expressing mature GCs and calretinin (CRT)-expressing mossy cells in the hilus, but not in doublecortin (DCX)- and CRT-expressing immature neurons and GAD67-expressing GABAergic interneurons. Arrowheads, cells expressing a specific neuron-type marker but not EGFP. Asterisks, cells co-labeled with CRT and EGFP. ml: molecular layer, gcl: granule cell layer. Scale bars, 50 µm.

**
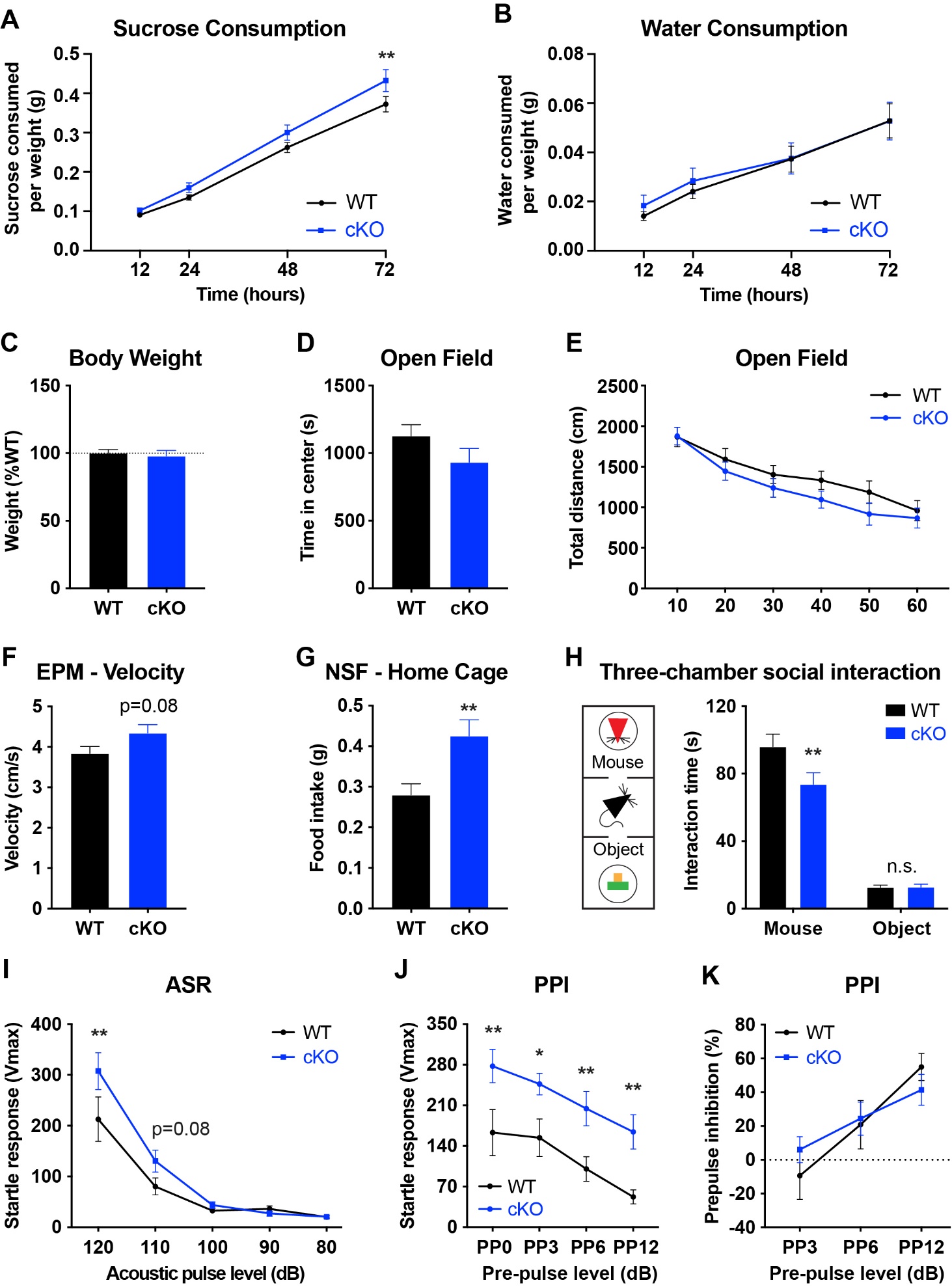
Fig. S3 | Additional behavioral assessment of cKO mice.** Related to Fig. 3. **a**, Quantification of the amount of 1% sucrose solution consumed over 72 hours normalized to body weight for KI198^Cre^::Htr4^loxP/loxP^ (cKO) mice and Htr4^loxP/loxP^ control littermates (WT) during the sucrose consumption test (SCT). Data were analyzed by repeated measures (RM) two-way ANOVA: time × genotype interaction, F(3,90) = 2.879, p = 0.0403; genotype factor, F(1,30) = 2.931, p = 0.0972 followed by post hoc Fisher’s LSD test, **p = 0.0077. n_WT_ = 16, n_cKO_ = 16. **b**, Quantification of water consumption during the SCT for each genotype. RM two-way ANOVA: time × genotype interaction, F(3,90) = 0.4320, p = 0.7306; genotype factor, F(1,30) = 0.09834, p = 0.7560. n_WT_ = 16, n_cKO_ = 16. **c**, Body weight of the mice of each genotype at the end of the SCT. Two-tailed unpaired t-test, p = 0.682. n_WT_ = 16, n_cKO_ = 16. **d**, Time spent in the center in the OF test over 60 min. Two-tailed unpaired t-test, p = 0.1443. n_WT_ = 20, n_cKO_ = 17. **e**, Quantification of locomotor activity (total distance traveled) for each 10-min bin in the OF test. RM two-way ANOVA: genotype factor, F(1,35) = 0.9715, p = 0.3311. n_WT_ = 20, n_cKO_ = 27. **f**, cKO mice showed a trend towards higher velocity (cm/s) in the EPM compared to WT. Two-tailed unpaired t-test, p = 0.0761. n_WT_ = 19, n_cKO_ = 16. **g**, Home cage food intake was measured for 30 min following the NSF. Two-tailed unpaired t-test, **p = 0.0044. n_WT_ = 20, n_cKO_ = 16. **h**, Quantification of time spent interacting with an unfamiliar mouse (Mouse) or a novel inanimate object (Object) in the three-chamber social interaction test for cKO or WT controls. Schematic of the testing apparatus is shown at left. RM two-way ANOVA: genotype factor, F(1,44) = 4.572, p = 0.0381 followed by post hoc Fisher’s LSD test, **p = 0.0038; ^n.s.^p = 0.9750. n_WT_ = 12, n_cKO_ = 12. **i**, cKO mice displayed larger acoustic startle responses (ASR) compared to WT to increasing levels of acoustic pulses. dB, decibel. RM two-way ANOVA: dB × genotype interaction, F(4,84) = 2.618, p = 0.0407; genotype factor, F(1,21) = 3.409, p = 0.0790 followed by post hoc Fisher’s LSD test, **p = 0.0012. n_WT_ = 11, n_cKO_ = 12. **j**, During the pre-pulse inhibition (PPI) test, cKO mice consistently exhibited higher startle responses to 120 dB acoustic stimuli preceded by any pre-pulse (PP) magnitude. RM two-way ANOVA: genotype factor, F(1,21) = 10.20, p = 0.0044 followed by post hoc Fisher’s LSD test, **p < 0.01, *p < 0.05. n_WT_ = 11, n_cKO_ = 12. **K**, cKO mice displayed similar levels of PPI as WT. RM two-way ANOVA: genotype factor, F(1,21) = 0.02151, ,p = 0.8848. n_WT_ = 11, n_cKO_ = 12. All data are presented as mean ± SEM.

**
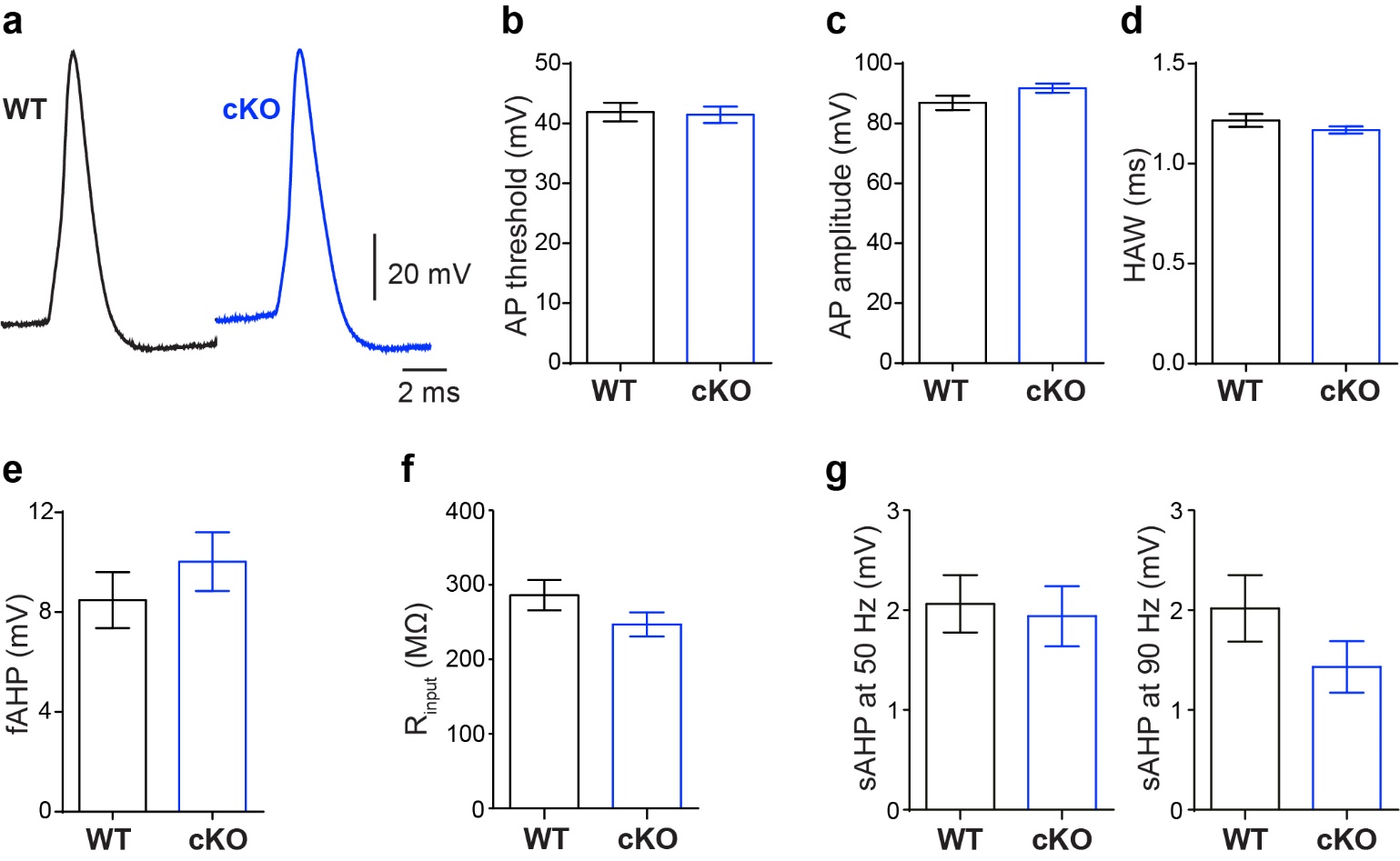
**

**Fig. S4 | Effect of the loss of 5-HT_4_R on the baseline electrophysiological properties of DG GCs.** Related to Fig. 3. **a**, Representative single action potentials (APs) from WT (black) and cKO (blue) DG GCs. **b-e**, No significant differences in AP properties were observed between genotypes: AP threshold, (**b**); AP amplitude (**c**), half-amplitude width, HAW (**d**); fast afterhyperpolarization potential, fAHP (**e**). **f**, Input resistance (R_input_) was not different between genotypes. **g**, Slow afterhyperpolarization potential (sAHP) was similar between genotypes after 1 s trains of AP at both 50 Hz (left) and 90 Hz (right). All data are presented as mean + SEM. For both genotypes, 11 neurons from three mice were used for each experiment. Two-tailed unpaired t-test was used for all statistical analyses.

**
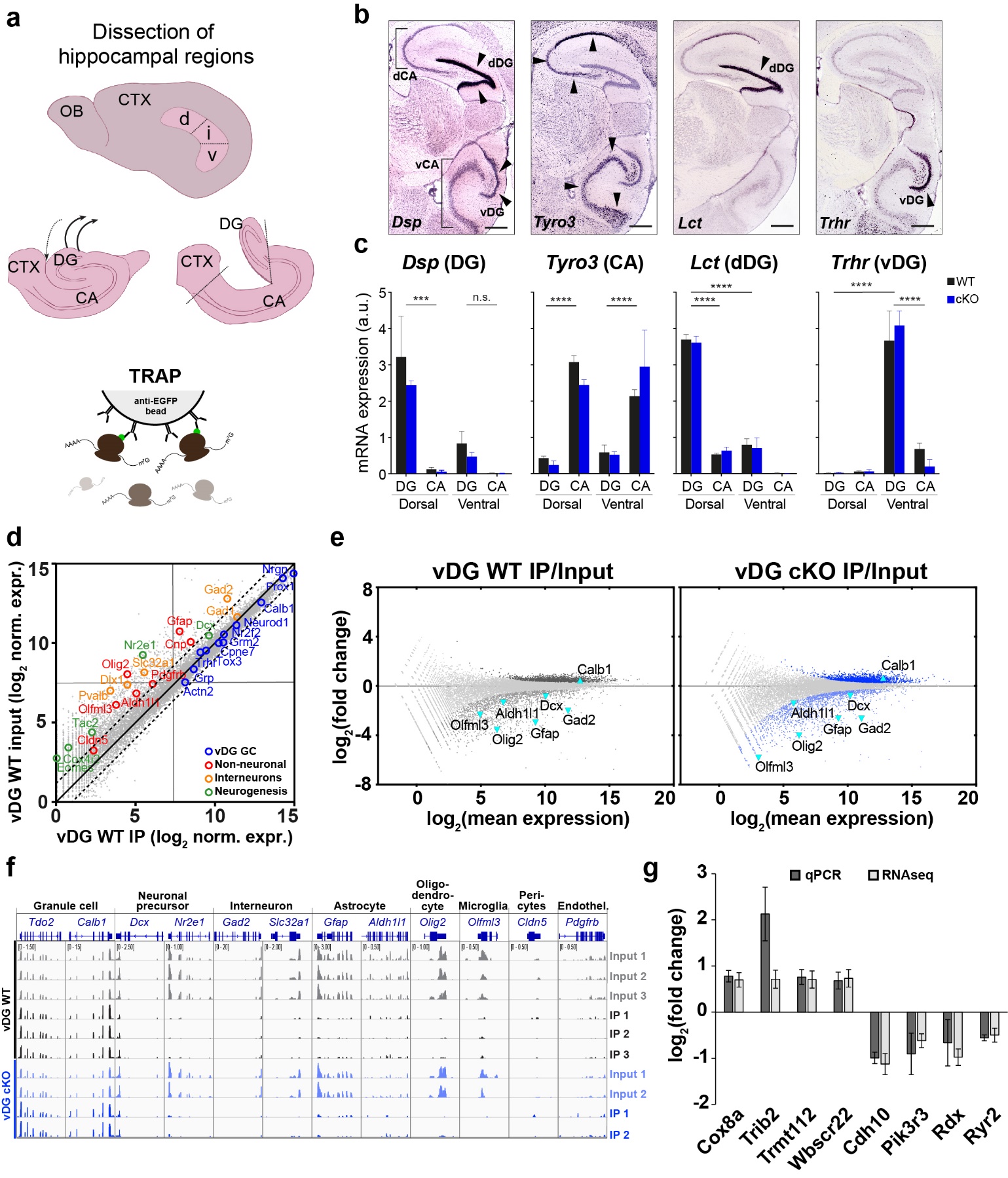
Fig. S5 | Assessment of regional and cell type specificity of vDG TRAP samples.** Related to Fig. 4. **a**, Schematic representation of the dissection of hippocampal regions. **b**, In situ hybridization (ISH) on sagittal sections through hippocampus showing the expression of region-specific genes. dDG: dorsal dentate gyrus, dCA: dorsal CA fields, vDG: ventral dentate gyrus, vCA: ventral CA fields. Arrowheads show region-specific expression for each gene. ISH images are © 2007 Allen Institute for Brain Science and available from: <http://www.mouse.brain-map.org>. **c**, qRT-PCR (mean ± SEM) showing relative expressions of region-specific genes from b in whole tissue mRNA samples. Two-way ANOVA followed by post hoc Fisher’s LSD test comparing region means. *Dsp:* genotype × region Interaction, F(3,12) = 0.2140, p=0.8848; genotype factor, F(1,12) = 0.6292, p = 0.4431; region factor, F(3,12) = 12.10, p = 0.0006; post hoc, ***p=0.0003, ^ns^p = 0.2501. *Tyro3*: genotype × region Interaction, F(3,12) = 1.916, p = 0.1809; genotype factor, F(1,12) = 0.005819, p=0.9405; region factor, F(3,12) = 34.25, p < 0.0001; post hoc, ****p < 0.0001. *Lct*: genotype × region interaction, F(3,12) = 0.2134, p = 0.8852; genotype factor, F(1,12) = 0.05478, p = 0.8189; region factor, F(3,12) = 279.0, p < 0.0001; post hoc, ****p < 0.0001. *Trhr*: genotype × region interaction, F(3,12) = 0.4318, p=0.7340; genotype factor, F(1,12) = 0.002429, p=0.9615; region factor, F(3,12) = 44.02, p < 0.0001; post hoc, ****p < 0.0001. **d**, Scatterplot comparing gene expression between input and IP in the vDG. Cell type marker genes are highlighted as vDG GC-specific (blue), non-neuronal (red), interneuron specific (yellow) and neurogenic (green). **e**, MA-plots showing differential expression analysis between IP and input samples from the vDG of WT (left) and cKO (right). Differentially expressed genes (FDR < 0.05) colored. Selected cell type marker genes are labeled. **f**, Genome browser view of RNA-seq reads mapped to selected cell type marker genes shows that IP samples, compared to input counterparts, were enriched in granule cell specific genes (*Tdo2*, *Calb1*), and depleted for genes specific for neuronal precursors (*Dcx*, *Nr2e1*), interneurons (*Gad2, Slc32a1*), astrocytes (Gfap, *Aldh1l1*), oligodendrocytes (Olig2), microglia (*Olfml3*), pericytes (*Cldn5*) and endothelial cells (*Pdgfrb*). **g**, Secondary confirmation of selected differentially expressed genes between WT and cKO vDG TRAP IPs by quantitative RT-PCR (dark gray bars). Values from RNAseq analysis are plotted for comparison (light gray bars). All data are mean + SEM.

**
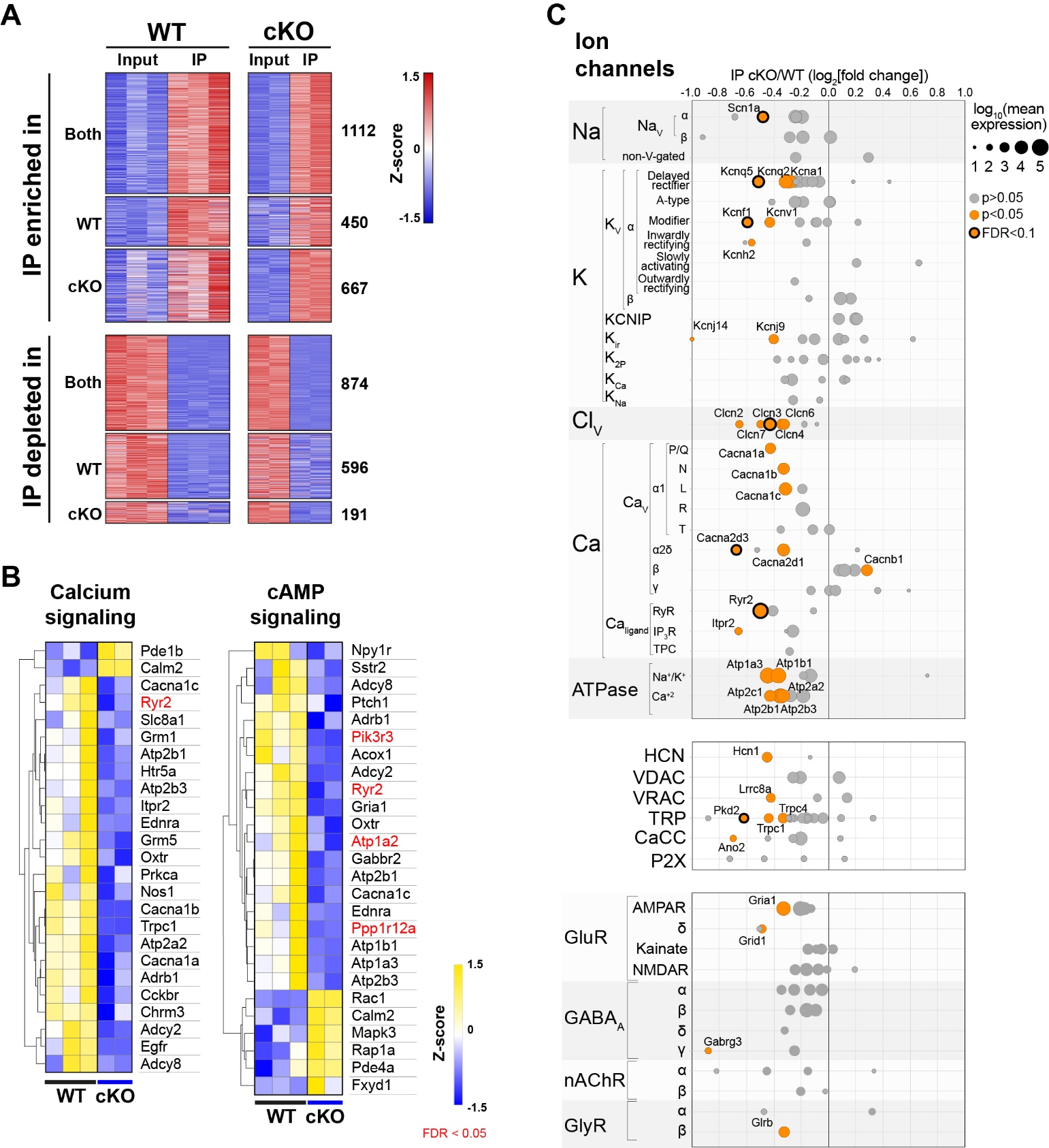
Fig. S6 | Genes related to 5‑HT_4_R signaling and neuronal function were altered following 5‑HT_4_R loss.** Related to Fig.4. **a**, Heatmap comparing normalized expression of IP enriched and depleted genes for each genotype in each sample. Detailed gene lists and values can be found in **Table S2**. **b**, Heatmap showing normalized expression values of calcium and cAMP signaling pathway-related genes in each TRAP IP mRNA from each genotype. Only genes with p < 0.05 are represented, red gene symbols indicate FDR < 0.05. **c**, Differential expression of ion channels in TRAP IP cKO/WT (log_2_[fold change]). Ion channels with normalized expression < 10 in the TRAP IP were excluded. Size of the bubbles are based on normalized mean expression of each gene across genotypes.

**
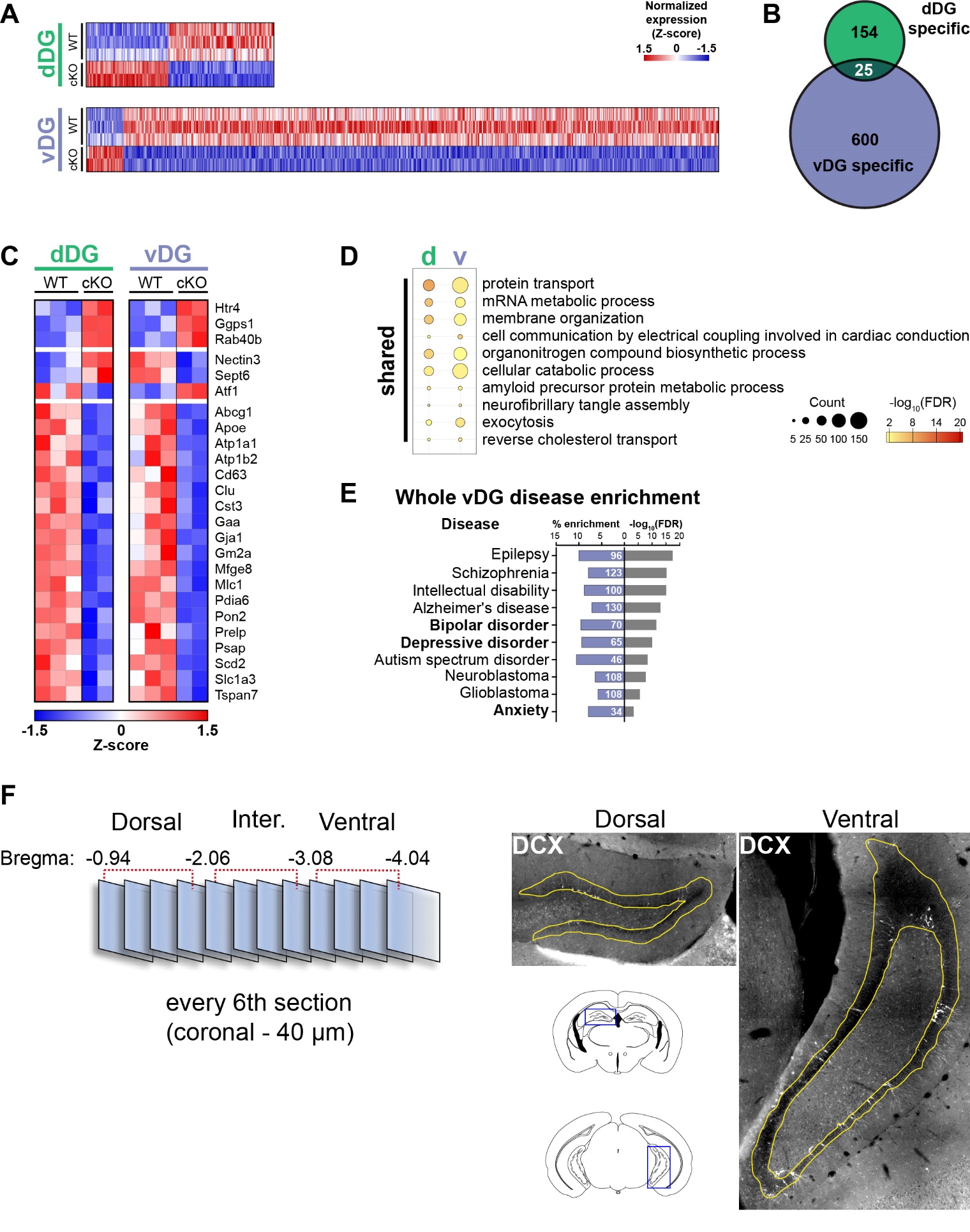
Fig. S7 | Comparison of the molecular properties of the dDG and vDG following 5‑HT_4_R loss.** Related to Fig. 5. **a**, Heatmaps of normalized expression values showing differentially expressed (DE) genes (FDR < 0.05) between WT and cKO in the whole tissue from dDG (top) and vDG (bottom). **b**, Venn diagram showing the number of overlapping DE genes between WT and cKO in the two regions. **c**, Heatmap showing 25 genes that were significantly altered in the cKO in both the dDG and vDG. Note that three genes (*Nectin3*, *Sept6,* and *Atf1*) were regulated in the opposite directions between two regions. **d**, Summary of shared gene ontology (GO) terms enriched in dDG and vDG (related to **Fig. 5b**). **e**, Summary of disease enrichment analysis of the DE genes in the vDG. For complete list, see **Table S9**. Note that bipolar disorder, depressive disorder and anxiety were among the significantly enriched diseases. No diseases showed significant association in DE genes of the dDG. **f**, Schematic depicting the experimental approach for quantification of neurogenesis. Left, the hippocampus was divided into three subdivisions (dorsal, intermediate, ventral) depending on the relative position of each coronal section to bregma. DCX+ cells were counted for every sixth 40 μm section. Right, example images of dorsal and ventral DG showing the area (highlighted in yellow) used for analysis. See **Supplemental Methods** for details.

**ADDITIONAL SUPPLEMENTAL FILES:**

**Table S1. vDG IP cKO vs. WT DE genes.** Related to Fig. 4. List of differentially expressed (DE) genes between cKO and WT in vDG IP samples.

**Table S2. vDG IP vs. input enriched and depleted genes.** Related to Figs. 4 and S6. List of genes enriched and depleted in the IP compared to input for both WT vDG and cKO vDG samples.

**Table S3. vDG IP GSEA.** Related to Fig. 4. Gene set enrichment analysis results for vDG IP data sets.

**Table S4. vDG IP GO analysis.** Related to Fig. 4. Gene ontology analysis results for DE genes between genotypes in vDG IP samples.

**Table S5. dDG input cKO vs. WT DE genes.** Related to Figs. 5 and S7. List of DE genes between cKO and WT in dDG input samples.

**Table S6. vDG input cKO vs. WT DE genes.** Related to Figs. 5 and S7. List of DE genes between cKO and WT in vDG input samples.

**Table S7. dDG and vDG input GO analysis.** Related to Figs. 5 and S7. Gene ontology analysis results for DE genes between genotypes for both dDG input and vDG input.

**Table S8. vDG input only GO analysis.** Related to Fig. 5. Gene ontology analysis results for whole vDG (input)-specific DE genes.

**Table S9. vDG input disease enrichment.** Related to Figs. 5 and S7. Disease enrichment analysis results for DE genes between genotypes for vDG input.

**Document S1. *Htr4* Targeting Vector (TV).** Related to Figs. 1 and S1. Annotated TV sequence for the generation of the Htr4^Floxed^ line.

**SUPPLEMENTARY METHODS:**

**Animals**

All procedures involving animals were approved by The Rockefeller University Institutional Animal Care and Use Committee and were in accordance with National Institutes of Health guidelines. KI198^Cre^ (Drd3-Cre KI198) mice were generated by the GENSAT Project (1) and were purchased from the Mutant Mouse Regional Resource Center (MMRRC) Repository (Stock #031741-UCD). Rosa26^fsTRAP^ mice were purchased from the Jackson Laboratories (Stock #022367). Htr4^Floxed^ and Htr4-bacTRAP mice were generated as described below. All animals were bred on a C57BL/6J background, group-housed, maintained on a 12-hr light-dark cycle at The Rockefeller University, and given ad libitum access to food and water. Animals used in the study were male, except for RNA sequencing experiments for which samples from male and female mice were pooled.

*Generation of Htr4^Floxed^ mouse line.* Targeting vector (TV) was designed in collaboration with inGenious Targeting Laboratory Inc., Ronkonkoma, NY. For annotated TV sequence (in full), see **Document S1**. A 7.92 Kb region used to construct the targeting vector (TV) was first subcloned from a positively identified C57BL/6 BAC clone (RP23:358G18) into a ~2.4kb backbone vector (pSP72, Promega, Madison, WI) containing an ampicillin selection cassette for retransformation of the construct prior to electroporation. A pGK-gb2 loxP/FRT neomycin resistance (Neo) cassette was inserted into the gene as depicted in **Fig. S1a**. The region was designed such that the short homology arm (SA) extended 2347 base pairs (bp) 3’ to the Neo cassette. The long homology arm (LA) ended 5’ to the target region and was 4820 bp long. The loxP/FRT flanked Neo cassette was inserted 252 bp downstream of exon 5. The single loxP site, containing engineered MfeI and ApaI sites for southern blot analysis, was inserted 347 bp upstream of exon 5. The target region was 752 bp and included exon 5. The total size of the targeting construct (including vector backbone and Neo cassette) was 13.69 Kb. The targeting vector was confirmed by restriction analysis after each modification step, and finally, by sequencing with the following primers: P6 (5’- GAG TGC ACC ATA TGG ACA TAT TGT C -3’), T73 (5’- TAA TGC AGG TTA ACC TGG CTT ATC G -3’), N1 (5’- TGC GAG GCC AGA GGC CAC TTG TGT AGC -3’), and N2 (5’- TGC GAG GCC AGA GGC CAC TTG TGT AGC -3’). The P6 and T73 primers anneal to the backbone vector sequence and read into the 5’ and 3’ ends of the BAC sub-clone and the N1 and N2 primers anneal to the 5’ and 3’ ends of the loxP/FRT flanked Neo cassette and sequence the 5’ side of SA and 3’ side of the target region, respectively. The TV was linearized using Notl prior to electroporation into C57BL/6 mouse embryonic stem cells. Three lines of chimeric mice were generated at the Janelia Research Campus Gene Targeting and Transgenic Facility, Ashburn, VA. One of these lines (1G12) showed germline transmission of the mutation. The 1G12 line was crossed to a germline Flpe recombinase line to remove the Neo cassette, leaving only two loxP sites flanking exon 5 of *Htr4*. We established this subsequent line, which are now referred to as “Htr4^Floxed^ mice.”

*Generation of Htr4-bacTRAP mouse line.* To generate the Htr4-bacTRAP mice, the RP23-53D24 BAC, which contained the *Htr4* locus, was modified using the two-plasmid/one recombination protocol as described previously (2,3). Briefly, a homology arm corresponding to the region immediately upstream of the ATG translation initiation site of the *Htr4* gene was cloned into the pS296 targeting vector (4) containing EGFPL10a using the AscI and NotI restriction sites. Recombination was performed by electroporating the pS296-Htr4 vector into electrocompetent DH10β bacteria containing pSV1.RecA plasmid and the BAC. Successful recombination was determined by screening cointegrates by PCR and Southern blot analysis of HindIII digested BAC DNA, using the homology region as a probe. The modified BAC was prepared by double acetate purification with CsCl centrifugation followed by membrane dialysis and microinjected into the pronuclei of fertilized FVB/N mouse oocytes at a concentration of 0.5 ng/µl. Five transgenic founder mice were generated and crossed to C57BL/6J mice. F1 progeny were screened for proper transgene expression by EGFP immunohistochemistry. Founder line ES1299 showed accurate and robust expression of the transgene and was therefore selected for colony expansion.

*Genotyping.* At age 15-18 days, pups were marked with an ear punch for identification and tail biopsies were performed. Each tail sample was lysed for DNA extraction in 200 µl tail lysis buffer (100 mM Tris.HCl-pH8.8, 1 mM EDTA, 0.5% Tween20, 100 ug/mL proteinase K) at 56 °C for at least 24 hours. The tail DNA samples were analyzed via PCR using 1-2 µl of lysis solution, GoTaq DNA Polymerase (Promega) and corresponding primer pairs as listed below.

Genotyping primers.

| **Mouse line** | **Primer*** | **Sequence** | **Amplicon**  **size (bp)** |
| --- | --- | --- | --- |
| Htr4-floxed | Htr4 loxP F | TCGAGGCATTCCTCGATTCA | WT: 172  loxP: 239 |
| Htr4-floxed | Htr4 loxP R | TAACACCTGGCCGAAACGTT |  |
| Any Cre | Cre F | CCGGGCTGCCACGACCAA | WT: none  Cre: 150 |
| Any Cre | Cre R | GGCGCGGCAACACCATTTTT |  |
| Any EGFPL10a | EGFP F | GCACGACTTCTTCAAGTCCGCCATGCC | WT: none  EGFP: 265 |
| Any EGFPL10a | EGFP R | GCGGATCTTGAAGTTCACCTTGATGCC |  |
| Rosa^fsTRAP^ | Rosa WT F | AAGGGAGCTGCAGTGGAGTA​ | WT: 297 |
| Rosa^fsTRAP^ | Rosa WT R | GAGCGGGAGAAATGGATA G |  |
| *F: forward, R: reverse | | | |

**cAMP induction assay**

Intact *Htr4* (WT) and mutant *Htr4^delE5^* (delE5) cDNA coding sequences (CDSs) (inserts) were cloned into pCMV6-Entry-Myc-DDK tagged mammalian expression vector (CAT#: PS100001, OriGene, Rockville, MD) by homologous recombination-based In-fusion HD cloning kit (Clontech, Takara Bio USA, Inc, Mountain View, CA). The inserts were amplified by PCR using CloneAmp HiFi PCR Premix (Clontech) with primers “Htr4 infusion forward” (5’- AGA TCT GCC GCC GCG ATC GCC ATG GAC AAA CTT GAT GCT AAT GTG -3’) and “Htr4 infusion reverse” (5’- GCG GCC GCG TAC GCG TAG TAT CAC TGG GCT GAG CAG -3’) from the cDNA samples generated from the dentate gyrus of either KI198^Cre^::Htr4^loxP/loxP^ (cKO) mice for delE5, or Htr4^loxP/loxP^ mice for WT expression. The primers were designed using “Online In-Fusion Tools” (<https://bit.ly/1eFKzsP>) to include 5’ and 3’ homologous sequences to the ends of the linearized vector for In-Fusion HD cloning, and the *Htr4* CDS start codon, yet exclude the stop codon to allow for the expression of the MYC-DDK tag sequences. pCMV6-EGFP was used as the control. HEK293T cells (ATCC, Manassas, VA) cultured in 24-well plates (1.9 cm^2^ culture area, Corning Inc, Corning, NY) in Dulbecco's Modified Eagle's Medium (DMEM) (Gibco, Thermo Fisher Scientific, Waltham, MA) supplemented with 10% fetal bovine serum (FBS) (Gibco) were transfected with the delE5, WT or EGFP plasmids using FuGENE 6 transfection reagent (Promega). Forty-eight hours after the transfection, all cells were incubated in stimulation buffer consisting of DMEM + 0.5mM IBMX (Sigma-Aldrich, St. Louis, MO) for 30 min. For cAMP induction, cells were further incubated in either stimulation buffer or 100µM Zacopride (Tocris Bioscience, Bio-Techne, Minneapolis, MN) in stimulation buffer for 45 min. The cells were then lysed with lysis buffer (0.1 M HCl, 0.1%Triton-X) for 20 min, and the lysates were centrifuged for 10 min at 21,500 x g. cAMP levels in the supernatants were measured by monoclonal anti-cAMP antibody based direct cAMP ELISA kit according to the manufacturer’s guidelines (Non-acetylated version, NewEast Biosciences, King of Prussia, PA). Four biological replicates and two technical replicates were performed for each experimental group. The cAMP level of each biological replicate was normalized to its mean protein concentration measured using Qubit protein assay kit (Invitrogen, Thermo Fisher Scientific) and Qubit 3.0 Fluorometer (Invitrogen). Statistical analysis of normalized cAMP levels was performed in GraphPad Prism 7 using one-way ANOVA followed by Fisher’s LSD.

**Immunohistochemistry**

For fixed brain serial slice preparations, mice were deeply anesthetized by ketamine/xylazine and transcardially perfused with 10 ml of phosphate buffered saline (PBS) followed by 30 ml of 4% paraformaldehyde (PFA) in PBS. Brains were post-fixed in 4% PFA overnight at 4°C and cryoprotected by 30% sucrose in PBS. Coronal sections (40 µm) were acquired using a freezing microtome (SM2010 R Sliding Microtome, Leica Biosystems Inc., Buffalo Grove, IL). The sections were then transferred to PBS, or cryoprotection buffer (25% glycerol and 25% ethylene glycol in PBS, pH 7.4) for long term storage at -20°C. For both 3,3′-Diaminobenzidine (DAB) immunohistochemistry (IHC) and all immunofluorescence (IF) procedures, free-floating serial sections were blocked for 60 min at room temperature in PBS containing 3-5% normal donkey serum (NDS, Jackson ImmunoResearch Laboratories, Inc., West Grove, PA) or normal goat serum (NGS, Vector Laboratories, Inc., Burlingame, CA) and 0.1% Triton X-100. Sections were then incubated in the same blocking buffer overnight at 4°C with primary antibodies listed below at corresponding dilutions. On the next day, the sections were washed three times with PBS each for 5 min, and then incubated for 2 h at room temperature with the appropriate Alexa dye-conjugated (for IF) or horseradish peroxidase (HRPT)-conjugated (for DAB IHC) secondary antibodies (Invitrogen) diluted 1:500 in the blocking buffer. The sections were then washed three times with PBS each for 5 min. For IF, the sections were counterstained with DAPI for nuclear staining at 1:10000 dilution followed by three times PBS wash each for 5 min. For DAB IHC, the staining was developed using SIGMAFAST™ DAB tablets (Sigma-Aldrich) following the manufacturer’s protocol. The sections were mounted on the Superfrost slides (VWR) and sealed with Prolong Gold mounting reagent (Invitrogen). The slides were imaged on a Zeiss LSM700 confocal microscope for **Fig. 2a-d**, **Fig.** **S2c,d** and **Fig.** **6e**, or a Zeiss Axioskop 2 microscope for **Fig. 1b**, **Fig.** **S2a,b** and **Fig.** **S6f**. Brightness was optimized using ImageJ software post-acquisition.

Primary antibodies used in this study.

| **Antibody Name** | **Species** | **Source** | **Catalog #** | **Dilution** |
| --- | --- | --- | --- | --- |
| Myc tag (9E10) | Monoclonal | Abcam | ab32 | 1:1000 |
| GAD67, clone 1G10.2 | Monoclonal | Millipore | MAB5406 | 1:500 |
| GFP | Chicken | Abcam | ab13970 | 1:1000 |
| NeuN, clone A60 | Monoclonal | Millipore | MAB377 | 1:1000 |
| Calbindin-D-28K | Rabbit | Sigma | C7354 | 1:500 |
| Calretinin | Monoclonal | Swant | 6B3 | 1:500 |
| Doublecortin (C-18) | Goat | Santa Cruz | sc-8066 | 1:200 |

**Quantification of neurogenesis**

An experimenter blinded to genotype counted number of DCX-positive (DCX+) cells in the DG granule cell layer (GCL) in every sixth 40 µm thick coronal sections through the entire hippocampus of WT or cKO mice (n = 5 per genotype). One hemisphere per animal was selected based on section quality and cells were counted at any depth through the entire section. Full size images of each section were then acquired and the area of GCL was calculated for each section by manually tracing the perimeter of the GCL as region of interest (ROI) in ImageJ. The hippocampus was divided into three subdivisions depending on the position of the coronal sections from bregma (5): dorsal (-0.96 to -2.06), intermediate (-2.06 to -3.08) and ventral (-3.08 to -4.04). Each tissue section was registered to a subdivision (4 ± 1 sections per animal per subdivision). Neurogenesis was calculated as the number of DCX+ cells per area of GCL within a subdivision and values for each animal (cKO and WT) was normalized to the WT mean for each subdivision.

**Fluorescent in situ hybridization (FISH)**

Mice were transcardially perfused first in PBS and then 4% paraformaldehyde in PBS. After dissection, brains were post-fixed overnight at 4°C . They were then cryopreserved in 30% sucrose and sectioned using a cryostat (14 µm sections). Antigen retrieval was performed on fixed frozen tissue using an Oster Steamer, followed by FISH (RNAscope® Technology). The RNAscope® Multiplex Fluorescent Reagent Kit V2 (Advanced Cell Dagnostics, Newark, CA) was used to target *Htr4* RNA using RNAscope® Probe Mm-Htr4 (Advanced Cell Diagnostics). Both antigen retrieval and FISH were performed according to manufacturer’s guidelines. Slides were coverslipped and imaged on an LSM700 confocal microscope.

**Total RNA isolation and quantitative RT-PCR (qRT-PCR)**

Hippocampus tissue samples were dissected with fine tip forceps in ice-cold HBSS containing 2.5 mM HEPES-KOH (pH 7.4), 35 mM glucose, 4 mM NaHCO3 and total RNA was isolated using RNeasy Micro Kit (Qiagen, Germantown, MD) with on-column DNase digestion. RNA quantity was measured with a Nanodrop 1000 spectrophotometer (Thermo Fisher Scientific). cDNA samples were generated from 300-1000 ng of total RNA using qScript cDNA SuperMix (QuantaBio, Beverly, MA). All qRT-PCR experiments were performed on the LightCycler 480 System (Roche Diagnostics, Indianapolis, IN), using TaqMan assays (Applied Biosystems, Thermo Fisher Scientific) and LightCycler 480 Probes Master mix (Roche). TaqMan assays used in this study are listed below. Default cycling conditions were followed (pre-incubation: one cycle, 95°C, 5 min; amplification: 45 cycles, 95°C for 10 s, 60°C for 30 s, 72°C for 1 s; ramp rate: 4.4°C/s). 10-20 ng of cDNA were used for each qRT-PCR reaction and three technical replicates were run for every sample. The mean C_T_ for each technical replicate was used for the quantification. Data were normalized to *Gapdh* as the endogenous control by the comparative C_T_ () method (6). For confirmation of TRAP RNAseq data in **Fig. S5g**, q-RT-PCR was carried out as described on 10 µg amplified TRAP IP cDNA and data were normalized to *Actb* by the comparative C_T_ method.

TaqMan gene expression assays used for qRT-PCR.

| **Gene Symbol** | **Assay ID** | **Dye** |
| --- | --- | --- |
| *Htr4* (3-4) | Mm00434129_m1 | FAM |
| *Htr4* (4-5) | Mm01258807_m1 | FAM |
| *Htr4* (5-6) | Mm01258808_m1 | FAM |
| *Htr4* (6-7) | Mm01258809_m1 | FAM |
| *Actb* | Mm00607939_s1 | FAM |
| *Cdh10* | Mm01130944_m1 | FAM |
| *Cox8a* | Mm02342396_g1 | FAM |
| *Dsp* | Mm01351876_m1 | FAM |
| *Gapdh* | Mm99999915_g1 | FAM |
| *Lct* | Mm01285112_m1 | FAM |
| *Pik3r3* | Mm00725026_m1 | FAM |
| *Rdx* | Mm01177363_m1 | FAM |
| *Ryr2* | Mm01340015_m1 | FAM |
| *Trhr* | Mm00443262_m1 | FAM |
| *Trib2* | Mm00454876_m1 | FAM |
| *Trmt112* | Mm01617623_g1 | FAM |
| *Tyro3* | Mm00444547_m1 | FAM |
| *Wbscr22* | Mm00458592_m1 | FAM |

**Translating ribosome affinity purification (TRAP)**

Affinity purification of EGFP-tagged polysomes was performed as previously described (7), with minor modifications. KI198^Cre^::Rosa26^fsTRAP^ (WT) and KI198^Cre^::Htr4^Floxed^::Rosa26^fsTRAP^ (cKO) mice were sacrificed, followed by rapid dissection of dorsal and ventral dentate gyrus with fine tip forceps in ice-cold HBSS containing 2.5 mM HEPES-KOH (pH 7.4), 35 mM glucose, 4 mM NaHCO3, and 100 μg/ml cycloheximide. Tissue from one male and one female mouse was pooled for each biological replicate. Pooled dentate gyrus samples were then homogenized in 1 ml extraction buffer containing 10 mM HEPES-KOH (pH 7.4), 150 mM KCl, 5 mM MgCl2, 0.5 mM DTT, 100 μg/ml cycloheximide, RNasin (Promega) and SUPERas-In (Life Technologies, Thermo Fisher Scientific) RNase inhibitors, and Complete-EDTA-free protease inhibitors (Roche). Homogenates were cleared by centrifugation at 2000 x g at 4 °C. IGEPAL CA-630 (NP-40, Sigma) and DHPC (Avanti Polar Lipids, Alabaster, AL) were both added to the supernatants (S2) to a final concentration of 1% for each, followed by centrifugation at 20,000 x g. Polysomes were immunoprecipitated from these supernatants (S20) using 100 μg monoclonal anti-EGFP antibodies (50 μg each of clones 19C8 and 19F7 (4)) bound to biotinylated-Protein L (Pierce, Thermo Fisher Scientific) coated streptavidin-conjugated magnetic beads (Life Technologies), and washed in low salt buffer containing 10 mM HEPES-KOH (pH7.4), 150 mM KCl, 5 mM MgCl2, 1% IGEPAL CA-630, 0.5 mM DTT, 100 μg/ml cycloheximide, and RNasin RNase inhibitors (Promega). IPs were carried out overnight at 4°C and beads were washed with and washed in high salt buffer containing 10 mM HEPES-KOH (pH7.4), 350 mM KCl, 5 mM MgCl2, 1% IGEPAL CA-630, 0.5 mM DTT, 100 μg/ml cycloheximide, and RNasin RNase inhibitors (Promega). Bound RNA was purified using the RNeasy Micro Kit (Qiagen) with on-column DNase digestion. RNA was also purified from a fraction of the pre-IP S20 supernatant to serve as whole-tissue (input) samples. RNA quantity was determined using the Qubit RNA HS Assay kit (Invitrogen) and RNA quality was determined using Agilent 2100 Bioanalyzer with RNA 6000 Pico chips. Only samples with RNA integrity values ≥ 7.0 were used for RNA-seq.

**RNA-sequencing (RNA-seq)**

For each sample, 15 ng of purified RNA was converted to cDNA and amplified using the Ovation RNA-Seq System V2 Kit (NuGEN, Redwood City, CA) following manufacture’s guidelines. cDNA was fragmented to an average size of 250 bp using a Covaris C2 sonicator (Covaris, Inc., Woburn, MA) with the following parameters: intensity 5, duty cycle 10%, cycles per burst 200, treatment time 120 seconds. RNA-seq libraries were prepared from 10 μg amplified RNA using the TruSeq RNA Sample Preparation Kit v2 (Illumina, San Diego, CA) following manufacturer’s protocols and libraries were sequenced at The Rockefeller University Genomics Resource Center on the Illumina NextSeq 500 platform to obtain 75 bp paired-end reads. The RNA-seq datasets generated in this study are listed below.

Overview of RNA-seq datasets.

| **Sample*** | **FastQ Name** |
| --- | --- |
| vDG_WT_IP_1 | KI198-Cre_vDG_WT_IP_Rep1 |
| vDG_WT_IP_2 | KI198-Cre_vDG_WT_IP_Rep2 |
| vDG_WT_IP_3 | KI198-Cre_vDG_WT_IP_Rep3 |
| vDG_cKO_IP_1 | KI198-Cre_vDG_Htr4_cKO_IP_Rep1 |
| vDG_cKO_IP_2 | KI198-Cre_vDG_Htr4_cKO_IP_Rep2 |
| vDG_WT_Input_1 | KI198-Cre_vDG_WT_input_Rep1 |
| vDG_WT_Input_2 | KI198-Cre_vDG_WT_input_Rep2 |
| vDG_WT_Input_3 | KI198-Cre_vDG_WT_input_Rep3 |
| vDG_cKO_Input_1 | KI198-Cre_vDG_Htr4_cKO_input_Rep1 |
| vDG_cKO_Input_2 | KI198-Cre_vDG_Htr4_cKO_input_Rep2 |
| dDG_WT_Input_1 | KI198-Cre_dDG_WT_input_Rep1 |
| dDG_WT_Input_2 | KI198-Cre_dDG_WT_input_Rep2 |
| dDG_WT_Input_3 | KI198-Cre_dDG_WT_input_Rep3 |
| dDG_cKO_Input_1 | KI198-Cre_dDG_Htr4_cKO_input_Rep1 |
| dDG_cKO_Input_2 | KI198-Cre_dDG_Htr4_cKO_input_Rep2 |
| * brain region_genotype_RNA source_replicate | |

**RNA-seq read mapping, analysis, and visualization**

RNA-seq read quality was assessed by FastQC (0.11.4). Sequences were trimmed for trailing adaptors from the Illumina sequencing process by Trim Galor (0.4.1). The trimmed reads were then aligned to annotated exons using the mm10 mouse reference genome (UCSC) with STAR (2.4.2a) (9) using default settings. SAMtools (v0.1.19-444) was used for indexing and removal of duplicates. The numbers of raw and mapped reads (bases), and the percentage of mapping for each sample were calculated via Picard (1.123) using the CollectRNASeqMetrics program and listed below. The quantification of aligned, sorted and indexed reads was done using the htseq-count module of the HTSeq framework (0.6.0) (10), using the “union” mode with default settings to generate raw counts for each sample. Aligned bam files were converted to tdf format using igvtools (2.3.61) for Integrative Genomics Viewer (IGV) visualization. Differential expression analyses were performed using DESeq2 (11), R-package version 1.4.5. Differentially expressed (DE) genes for each analysis were determined based on adjusted p-values (p-adj), also termed as false discovery rate (FDR), and reported in Results or the figure legends. IP enriched genes were determined as IP over input fold change ≥ 1.25 with FDR < 0.05, and IP depleted genes as IP over input fold change < 0.5 with FDR < 0.05. For **Fig. 4d-f**, when determining DE genes between the cKO and WT TRAP mRNA, nine genes were detected as genes normally depleted in IP (IP depleted in WT) and excluded from further analysis (red genes in Table S2). For MA-plots, log_2_ of the fold change between genotypes (log_2_[fold change]) and log_2_ of the mean of the normalized expression values in all samples (log_2_[mean expression]) for each gene were used. Scatter plots were generated using normalized expression values (log_2_[norm. expr.]). Heatmap visualizations (one minus Pearson correlation) of normalized expression values were generated using web-based Morpheus software (Broad Institute, <https://software.broadinstitute.org/morpheus)> and z-scores were calculated using mean subtracted and SD normalized values by gene. Nominal p-values (p) were also given when normalized expressions of specific genes were reported. Full lists of DE genes can be found in **Tables S1** (vDG IP cKO vs. WT), **S2** (IP vs. input for cKO and WT), **S5** (dDG input cKO vs. WT), and **S6** (vDG input cKO vs. WT). Gene set enrichment analysis (GSEA) was performed on the fold change-ranked gene list of IP samples comparing cKO and WT (p < 0.05, log[norm. expr.] > 1). We also excluded genes strongly depleted in the IP (WT IP/Input enrichment FDR < 0.05, log_2_[fold change] < -1) and *Htr4* as GSEA accounts for the direction of the regulation of gene expression and *Htr4* expression seemed upregulated although it was non-functional. GSEA desktop v3.0 software (Broad Institute) was used with the following parameters: Gene sets database = Molecular Signature Database (MSigDB), C2, Canonical Pathways (CP, c2.cp.v6.2.symbols.gmt); number of permutations = 1000; enrichment statistics = classic; set size, max = 100, min = 10; normalization = meandiv. Gene sets with FDR ≤ 25 are reported as advised (12). Gene ontology (GO) and disease enrichment analyses were performed using ToppGene Suite (<http://toppgene.cchmc.org>) (13). “GO: Biological Process” results were reported for GO analyses. FDR (Benjamini-Hochberg) < 0.05 was considered statistically significant. Network view in **Fig. 5h** was generated using the EnrichmentMap plugin (3.1.0) for Cytoscape (3.7.0) using enriched GO terms (FDR < 0.01, nodes) and the percentage of overlapping genes in among those terms (overlap > 50%, edges).

Quality control of RNA-seq alignments.

| **Sample** | **Raw**  **Reads** | **Mapped Reads** | **Riboso-mal%** | **Coding %** | **UTR %** | **Intro-**  **nic%** | **Interge-**  **nic %** | **mRNA* %** |
| --- | --- | --- | --- | --- | --- | --- | --- | --- |
| dDG_WT_Input_1 | 4976974951 | 4898777541 | 11.00 | 26.15 | 34.18 | 11.65 | 17.48 | 60.33 |
| dDG_WT_Input_2 | 4114194145 | 4048376030 | 9.60 | 28.97 | 36.85 | 9.12 | 15.87 | 65.82 |
| dDG_WT_Input_3 | 6348807839 | 6241867530 | 13.52 | 30.73 | 36.91 | 7.14 | 12.29 | 67.64 |
| dDG_KO_Input_1 | 5318465410 | 5237452256 | 9.40 | 29.55 | 37.17 | 8.97 | 15.33 | 66.72 |
| dDG_KO_Input_2 | 4494618359 | 4433172922 | 7.08 | 30.48 | 37.47 | 8.40 | 16.89 | 67.95 |
| vDG_WT_Input_1 | 5274266386 | 5200347558 | 7.32 | 29.76 | 37.30 | 9.69 | 16.25 | 67.07 |
| vDG_WT_Input_2 | 5908160994 | 5817160131 | 8.02 | 31.64 | 36.97 | 8.64 | 15.09 | 68.61 |
| vDG_WT_Input_3 | 5200024888 | 5110416182 | 12.45 | 30.12 | 36.81 | 7.70 | 13.47 | 66.93 |
| vDG_KO_Input_1 | 5390357255 | 5295752262 | 11.57 | 27.52 | 35.93 | 8.45 | 17.04 | 63.45 |
| vDG_KO_Input_2 | 5347253361 | 5264487834 | 10.03 | 29.78 | 38.46 | 8.79 | 13.38 | 68.24 |
| vDG_WT_IP_1 | 5055676715 | 4963214738 | 15.25 | 35.93 | 39.45 | 3.22 | 6.83 | 75.37 |
| vDG_WT_IP_2 | 5200875692 | 5096531166 | 18.77 | 34.24 | 37.98 | 2.97 | 6.88 | 72.22 |
| vDG_WT_IP_3 | 5176647321 | 5088146609 | 11.74 | 39.38 | 38.93 | 3.07 | 7.41 | 78.31 |
| vDG_KO_IP_1 | 4386834142 | 4294720920 | 18.98 | 33.93 | 37.81 | 3.40 | 6.72 | 71.74 |
| vDG_KO_IP_2 | 5041820503 | 4938661168 | 18.97 | 34.77 | 37.26 | 3.27 | 6.56 | 72.04 |
| * mRNA is the percentage of total bases mapping to Coding + UTR. | | | | | | | | |

**Electrophysiological recordings**

Eight-week-old mice were euthanized with CO_2_. After decapitation and removal of the brain, transverse slices (400 μm thickness) were cut using a Vibratome 1000 Plus (Leica Biosystems) at 2 °C in a NMDG-containing cutting solution (in mM): 105 NMDG (N-Methyl-D-glucamine), 105 HCl, 2.5 KCl, 1.2 NaH_2_PO_4_, 26 NaHCO_3_, 25 Glucose, 10 MgSO_4_, 0.5 CaCl_2_, 5 L-Ascorbic Acid, 3 Sodium Pyruvate, 2 Thiourea (pH was around 7.4, with osmolarity of 295–305 mOsm). After cutting, slices were left to recover for 15 min in the same cutting solution at 35 °C and for 1 h at room temperature (RT) in recording solution (see below). Whole-cell patch-clamp recordings were performed with a Multiclamp 700B/Digidata1550A system (Molecular Devices, LLC., San Jose, CA). Dentate gyrus granule neurons were selected for recording based on their size, shape and position in the granular layer using an upright Olympus BX51WI microscope. The extracellular solution used for recordings contained (in mM): 125 NaCl, 25 NaHCO_3_, 2.5 KCl, 1.25 NaH_2_PO_4_, 2 CaCl_2_, 1 MgCl_2_ and 25 glucose (bubbled with 95% O_2_ and 5% CO_2_). The slice was placed in a recording chamber (RC-27L, Warner Instruments) and constantly perfused with oxygenated aCSF at 24 °C (TC-324B, Warner Instruments) at a rate of 1.5–2.0 ml/min. Whole-cell patch-clamp recordings were obtained from granule neurons using recording pipettes (Glass type 8250, King Precision Glass, Inc, Claremont, CA) pulled in a horizontal pipette puller (Narishige International USA, Inc., Amityville, NY) to a resistance of 3–4 MΩ, filled with an internal solution containing (in mM): 126 K-gluconate, 4 NaCl, 1 MgSO_4_, 0.02 CaCl_2_, 0.1 BAPTA, 15 glucose, 5 HEPES, 3 ATP, 0.1 GTP (pH 7.3). For whole-cell recordings in the voltage clamp configuration, BIMU-8 (10 mM, Tocris Bioscience) was added to the bath for 1 minute and subsequently washed. Tetradotoxin (TTX, 1 mM, Tocris Bioscience) was added previously to the bath to avoid activation of neighboring neurons by BIMU-8. To measure the firing of the DG granule neurons, steps of 10 pA current were injected from a set starting membrane potential of -80mV. Data were acquired at a sampling frequency of 50 kHz and filtered at 1 kHz and analyzed offline using pClamp10 software (Molecular Devices).

**Behavior**

Behavioral tests were performed on male mice from eight weeks up to four months of age. All mice were age-matched for each test, and control groups always consisted of WT littermates. For each test, mouse order was randomized, and the experimenter was blinded to genotype during the tests and data analyses. Mice were brought into the procedure room in their home cages and habituated to the room for one hour. Tests were performed within the light period of the light-dark cycle unless otherwise noted. The number of animals per group (n) and the statistical analyses are reported in the figure legends. For analysis of each behavioral tests, data points outside two standard-deviations of the mean for each group were determined as outliers and excluded. Statistical analyses were performed using GraphPad Prism 7 and 8 software, and p < 0.05 was considered significant.

*Tail suspension test.* Adhesive tape was used to suspend mice by their tail (0.5-1 cm from the tip of the tail) to the end of a piece of flexible plastic tubing hanging from the top center of one of the four compartments of a white acrylic box. Four mice were tested at the same time in each compartment and were separated by white acrylic walls. While suspending, mice were about 30 cm above the floor of the box. Test sessions lasted 6 min and were videotaped. Mice that held on to their bodies using their front limbs or started climbing up their tails were gently repositioned using a long stick without distracting other subjects. The floors and the walls of the box were cleaned with Clidox sterilizer and water in between trials. The amount of time spent immobile in the last 4 min of the test was quantified, which was extrapolated by scoring as mobile or immobile in every 5 s. In case of clasping to body parts or climbing up the tail, scoring was not applicable, and those mice were excluded from the analysis

*Forced swim test.* Mice were individually placed into glass cylinders (15 cm diameter, 35 cm height) filled with tap water (23-25 °C) to a height of 15.7 cm. Four mice were tested at the same time in separate cylinders placed as 2x2 adjacently. The cylinders were visually separated by white acrylic sheets. To start a trial, mice were gently placed into the cylinders keeping their head above the water surface. Test sessions lasted 6 min and were videotaped from above. The amount of time spent immobile, defined as the absence of all motions except floating and those required to keep a mouse’s head above the surface, was measured for the last 4 min of the trial, which was extrapolated by scoring as mobile or immobile in every 5 s.

*Splash test.* These experiments were performed in the dark cycle (20:00 – 23:59) to observe a higher amount of activity. The procedure room was illuminated with red light. Each mouse was sprayed with 200 µl 10% sucrose in tap water on their lower back using a 1 ml syringe with a needle tip attached. Immediately after, mice were placed in clean small mouse cages (22.2x30.80x16.24 cm) with bedding and filter top (no wire top). Four mice were tested at the same time in adjacent cages visually separated by white acrylic sheets. Test sessions lasted 5 min and were videotaped from the side. Grooming time was measured for a total 5 min, which was extrapolated by scoring as grooming or non-grooming in every 5 s.

*Sucrose consumption test.* Each mouse was placed in a clean cage with bedding and food, containing two water bottles, one filled with 1% sucrose in tap water and another filled with just tap water. Bottles were 50 ml Falcon conical tubes with sipper caps composed of 2.5 cm straight stainless steel ball-point sipper tubes (Ancare, Bellmore, NY), inserted into rubber stoppers. Consumption of sucrose solution and water was monitored for 72 hours by measuring the decrease in the weight of the bottles every 12 h. At the each 12-hour mark, the bottles were switched to avoid side preference. At the end of the test, animals were weighed and the amount of sucrose and water consumed (g) was normalized the weight per animal (g).

*Open field test.* Open field behavior was assayed in a square, acrylic arena (50x50x22.5 cm) with white floor and clear walls, equipped with two rows of infrared photocells placed 20 and 50 mm above the floor, spaced 31 mm apart. The procedure room was brightly and homogenously lit with fluorescent ceiling lamps. Each animal was placed in the open field arena for 60 min. Photocell beam interruptions were recorded on a computer using the Fusion software (Omnitech Electronics, Inc., Columbus, OH). The floors and the walls of the arenas were cleaned with Clidox sterilizer and water in between trials. The data was exported as time spent and distance traveled in total, peripheral and center areas in 10 min bins.

*Elevated plus maze.* The elevated plus maze consisted of two opposite open arms without sidewalls (35x5 cm) and two enclosed arms with black 14 cm high sidewalls (35x5x14 cm) connected by a common central platform (5 cm^2^). The entire plus-maze apparatus was elevated 30 cm off the ground. The experimental area was isolated from the experimenter with black curtains running down from the ceiling to the floor on four-sides and homogenously lit by ceiling mounted LED lamps on four sides, ensuring minimal shadows. The illumination was adjusted so the middle of each open arm was at 30-lux. Testing began by placing an animal on the central platform of the maze facing the same open arm. Trials lasted 5 min and recorded by a ceiling mounted camera connected to a computer. EthoVision XT 7.0 software (Noldus Information Technology Inc., Leesburg, VA) was used to track the mouse and record the total time spent in each arm, total distance traveled, and velocity. The maze was wiped with 30% Ethanol in between trials. Mice that fell off the open arms were excluded from the analysis.

*Novelty suppressed feeding.* Mice were food deprived for 24-hour prior to testing. Body weight was monitored to determine efficiency of deprivation. Testing was performed in a brightly lit arena similar to the open field (50x50x22.5 cm) where a food pellet (1.8 cm length of 1.58x0.95 cm diameter oval pellets) was placed in the center on a circular filter paper (20 cm diameter, Fisherbrand, Thermo Fisher Scientific). A white noise source was active throughout the habituation and testing. Four subjects were tested at the same time in adjacent arenas. The latency to bite the food within a 15 min trial was recoded. Following NSF, each animal was put in a clean vivarium cage for 30 min with food ad libidum after each trial. The weight of the food was measured before and after the 30 min test.

*Acoustic startle response and pre-pulse inhibition.* Startle and PPI testing were performed in a startle response systems (39×38×58 cm, SR-LAB system, San Diego Instruments, San Diego, CA) consisting of a non-restrictive clear Plexiglas cylinders (inner diameter 4 cm, length 13 cm) resting on a white Plexiglas platform and placed in a ventilated, sound-attenuated chamber. High frequency speakers, controlled by SR-LAB software, were mounted 33 cm above the cylinders and used to present all acoustic stimuli. Piezoelectric accelerometers mounted under the cylinders transduced movements of the animals which were digitized and stored by an interface and computer assembly. A dynamic calibration system was used to ensure comparable sensitivities across four separate chambers. The test mouse was placed into the startle chamber and allowed to acclimatize for 5 min. The time between each trial was 7-23 s. The trial consisted of four blocks. All sound intensities were presented in a pseudo-random order within a block. Beginning at startling stimulus onset, 65 consecutive 1-ms readings were recorded to obtain the peak amplitude of the animal's startle response (Vmax). Five seconds after each stimulus, another 65 ms were measured which constitutes the no-stimulus trials. Background noise was 65 dB. In the first block (pre-test), mice were presented with 120 dB acoustic stimuli (65 ms) for five trials. Vmax were recorded per trial and averaged per subject. In the second block (ASR), acoustic stimuli were presented in five different intensities (120, 110, 100, 90 and 80 dB, 65 ms), each for four trials. Vmax was recorded for each trial and averaged for each stimulus intensity per subject. In the third block (PPI), a 120 dB acoustic stimulus (40 ms) was preceded by four different pre-pulse (PP) intensities (0, 3, 6, 12 dB, 20 ms) for ten trials. The time between the prepulse and the pulse was 100 ms. Vmax was recorded for each trial and averaged for each prepulse intensity per subject. PPI was calculated as the percent decrease in startle response in PP3, PP6 and PP12 compared to PP0 per subject. In the fourth block (post-test), 120 dB stimuli (65 ms) were presented for five trials. Vmax was recorded per trial, averaged per subject, and compared to the pre-test responses to assess the habituation of ASR during the testing.

*Social interaction.* Social behavior was measured using the three-chamber social interaction test. A rectangular test arena (58.5x43x22.7 cm) was made of white acrylic and two inner walls divided it equally into three chambers. Each inner wall had an open middle section with a removable sliding door, allowing free access between the middle and the two side chambers when the doors were removed. The social interaction test was comprised of two parts: a habituation session and a trial session. During the habituation session, two empty wire cups were placed in the left and right chambers. The test mouse was placed in the center chamber and allowed to explore all three chambers for 10 min. Next, the doors to the side chambers were closed, and the test mouse was placed in the middle chamber for the trial session. A stranger mouse of the same sex/appearance and a novel inanimate object (plastic building toy) of similar size were placed inside the left and right wire cups in a random order to balance for side preference. Doors between chambers were opened to allow the test mouse to explore the three chambers freely for 10 min. During all sessions, the arena was videotaped from above and the interaction time with each cup, defined as sniffing or touching cup, was manually measured from the recorded videos. Stranger mice were habituated to the wire cups for 10 min each day for three days prior to testing. The arena and the wire cups were thoroughly cleaned with Clidox sterilizer and water between subjects.

**Statistical analysis**

All data analysis was performed using GraphPad Prism 7 and 8, Microsoft Excel or R (14). Statistical parameters including the exact value of n, precision measures (mean ± SEM) and statistical significance are reported within the Results or the figure legends. Data were determined to be statistically significant when p < 0.05 by two-way ANOVA (ordinary or repeated measures [RM]) followed by post hoc Fisher’s LSD test, one-way ANOVA followed by post hoc Fisher’s LSD test, or two-tailed unpaired t-test. All data met the assumptions of applied statistical tests (e.g. normally distributed for parametric tests). Statistical methods to analyze RNA-seq data are discussed in the “RNA-seq read mapping, analysis, and visualization” section**.**

**References**

1. Gong S, Doughty M, Harbaugh CR, Cummins A, Hatten ME, Heintz N, Gerfen CR (2007): Targeting Cre recombinase to specific neuron populations with bacterial artificial chromosome constructs. *Journal of Neuroscience* 27: 9817–9823.

2. Gong S, Kus L, Heintz N (2010): Rapid bacterial artificial chromosome modification for large-scale mouse transgenesis. *Nature Protocols* 5: 1678–1696.

3. Gong S, Yang XW, Li C, Heintz N (2002): Highly efficient modification of bacterial artificial chromosomes (BACs) using novel shuttle vectors containing the R6Kgamma origin of replication. *Genome Res* 12: 1992–1998.

4. Heiman M, Schaefer A, Gong S, Peterson JD, Day M, Ramsey KE, *et al.* (2008): A Translational Profiling Approach for the Molecular Characterization of CNS Cell Types. *Cell* 135: 738–748.

5. Paxinos G, Franklin KBJ (2008): *The Mouse Brain in Stereotaxic Coordinates*. Academic Press.

6. Schmittgen TD, Livak KJ (2008): Analyzing real-time PCR data by the comparative CT method. *Nature Protocols* 3: 1101–1108.

7. Heiman M, Kulicke R, Fenster RJ, Greengard P, Heintz N (2014): Cell type-specific mRNA purification by translating ribosome affinity purification (TRAP). *Nature Protocols* 9: 1282–1291.

8. Barrett T, Wilhite SE, Ledoux P, Evangelista C, Kim IF, Tomashevsky M, *et al.* (2013): NCBI GEO: archive for functional genomics data sets--update. *Nucleic Acids Res* 41: D991–5.

9. Dobin A, Davis CA, Schlesinger F, Drenkow J, Zaleski C, Jha S, *et al.* (2013): STAR: ultrafast universal RNA-seq aligner. *Bioinformatics* 29: 15–21.

10. Anders S, Pyl PT, Huber W (2015): HTSeq--a Python framework to work with high-throughput sequencing data. *Bioinformatics* 31: 166–169.

11. Love MI, Huber W, Anders S (2014): Moderated estimation of fold change and dispersion for RNA-seq data with DESeq2. *Genome Biol* 15: 550.

12. Subramanian A, Tamayo P, Mootha VK, Mukherjee S, Ebert BL, Gillette MA, *et al.* (2005): Gene set enrichment analysis: a knowledge-based approach for interpreting genome-wide expression profiles. *Proceedings of the National Academy of Sciences* 102: 15545–15550.

13. Chen J, Bardes EE, Aronow BJ, Jegga AG (2009): ToppGene Suite for gene list enrichment analysis and candidate gene prioritization. *Nucleic Acids Res* 37: W305–11.

14. R Core Team (2017). R: A language and environment for statistical computing. R Foundation for Statistical Computing, Vienna, Austria. http://www.R-project.org/.
